# Tumor evolution selectively inactivates the core microRNA machinery for immune evasion

**DOI:** 10.1038/s41467-021-27331-3

**Published:** 2021-12-01

**Authors:** Tian-Yu Song, Min Long, Hai-Xin Zhao, Miao-Wen Zou, Hong-Jie Fan, Yang Liu, Chen-Lu Geng, Min-Fang Song, Yu-Feng Liu, Jun-Yi Chen, Yu-Lin Yang, Wen-Rong Zhou, Da-Wei Huang, Bo Peng, Zhen-Gang Peng, Yong Cang

**Affiliations:** 1 Gene Editing Center, School of Life Science and Technology, ShanghaiTech University, Shanghai, China; 2 Center for Excellence in Molecular Cell Science, Chinese Academy of Sciences, Shanghai, China; 3 Life Sciences Institute, Zhejiang University, Hangzhou, Zhejiang China; 4 Oncology and Immunology Unit, WuXi Biology, WuXi AppTec (Shanghai) Co, Ltd, Shanghai, China; 5 University of Chinese Academy of Sciences, 100049 Beijing, China

**Keywords:** Immunosurveillance, Cancer immunotherapy, Tumour heterogeneity

## Abstract

Cancer cells acquire genetic heterogeneity to escape from immune surveillance during tumor evolution, but a systematic approach to distinguish driver from passenger mutations is lacking. Here we investigate the impact of different immune pressure on tumor clonal dynamics and immune evasion mechanism, by combining massive parallel sequencing of immune edited tumors and CRISPR library screens in syngeneic mouse tumor model and co-culture system. We find that the core microRNA (miRNA) biogenesis and targeting machinery maintains the sensitivity of cancer cells to PD-1-independent T cell-mediated cytotoxicity. Genetic inactivation of the machinery or re-introduction of *ANKRD52* frequent patient mutations dampens the JAK-STAT-interferon-γ signaling and antigen presentation in cancer cells, largely by abolishing miR-155-targeted silencing of suppressor of cytokine signaling 1 (SOCS1). Expression of each miRNA machinery component strongly correlates with intratumoral T cell infiltration in nearly all human cancer types. Our data indicate that the evolutionarily conserved miRNA pathway can be exploited by cancer cells to escape from T cell-mediated elimination and immunotherapy.

## Abstract (web summary)

Dysregulation of the microRNA machinery has crucial roles in cancer development. Here the authors show that inactivation of proteins involved in microRNA-mediated gene silencing, such as ANKRD52 or AGO2, confers resistance to T cell-mediated immune response in a preclinical cancer model.

## Introduction

Cancer immunotherapies such as immune checkpoint blockade (ICB) unleash T cell cytotoxicity against cancer cells and have significantly improved the perspective of cancer patients. However, a majority of cancer patients fail to benefit durably from immunotherapies, mostly due to the cancer-intrinsic accumulation of somatic mutations driving primary and acquired resistance to the treatment^1–3^. Two complementary approaches have been adopted to identify genetic elements that control cancer cell sensitivity to T cell-mediated cytotoxicity. One approach is to directly interrogate the genomics and transcriptomics of clinical tumor samples from patients exhibiting variable responses to immunotherapies^4–6^, but the inherent genetic variation between patient cohorts poses a great challenge to pinpoint immunotherapy-relevant drivers from passenger hotspot mutations^7,8^. The other approach is to screen CRISPR-Cas9-based guide RNA (gRNA) libraries that target either the whole genome using cancer cell and immune cell co-culture systems^9,10^, or focused gene sets using immunocompetent murine tumors^11,12^. However, in vitro screens are limited to a lack of tumor microenvironment, and the gRNA library coverage or selection often limits the pathological relevance of targets unraveled by in vivo screens.

Combined, these approaches have led to the discovery of a myriad of mechanisms underlying cancer susceptibility to or evasion from T cell attack. Among them, the most prominent regulators affect antigen presentation and interferon-γ (IFNγ) signaling (B2M, JAK1/2, SOCS1, PTPN2, APLNR, and ADAR1)^4,11,13–16^, in addition to chromatin remodeling (PBRM1, ARID2, KMT2D, and ASF1A)^5,9,12,17^, and TNFα and autophagy pathways^10,18^. Despite these advances, our understanding of the immune evasion mechanism remains incomplete to overcome recurrent clinical resistance. Evolving cancer cells could theoretically accumulate many more mutations that regulate their responses or resistance to tumor immunity^7,11^. It is therefore imperative to uncover novel immune evasion mechanisms in the dynamic tumor-immune microenvironment by untangling the genetic heterogeneity of cancer cells.

MicroRNAs (miRNAs) are small non-coding RNAs that bind complementary messenger RNAs (mRNAs) to repress gene expression and regulate essentially all cellular processes^19^. MiRNAs are generated by stepwise cleavage via double-stranded ribonuclease III enzymes DROSHA and DICER1 and loaded onto Argonaute (AGO) proteins to pair with target sequences on mRNAs^20^. The miRNA-mRNA pairing triggers casein kinase 1α (CK1α)-induced phosphorylation and dissociation of AGO2 from the active complex, whereas the ANKRD52-PPP6C phosphatase complex dephosphorylates AGO2 to restore its miRNA loading activity^21,22^. Recurrent mutations in these core miRNA machinery components are identified in many cancer types including neuroblastoma, Wilms tumor, ovarian cancer, and melanoma^23–28^. For example, mutations in or reduced expression of *DROSHA* and *DICER1* in tumors associate with advanced tumor stage and poor clinical outcome in cancer patients^29–31^. Additionally, ANKRD52 was found as a suppressor of tumor metastases, and reduced ANKRD52 levels are associated with late-stage lung cancer^22,32^.

Although mutations in or dysregulation of miRNA machinery characterize a sizeable patient subpopulation and play crucial roles in cancer development, whether these genetic alterations in cancer cells could contribute to immune evasion or resistance to ICB is still unclear. Here, we uncover an unexpected role of the core miRNA machinery in enabling cancer cell sensitivity to T cell-mediated cytotoxicity by untangling tumor heterogeneity during unbiased immune selection. We demonstrate that disruption of the machinery mitigates miR-155-targeted silencing of *SOCS1* in cancer cells, and consequently suppresses essential signaling for T cell-mediated anti-tumor immunity.

## Results

### Profiling of cancer heterogeneity selected by increasing host immunity

**Fig. 1:**
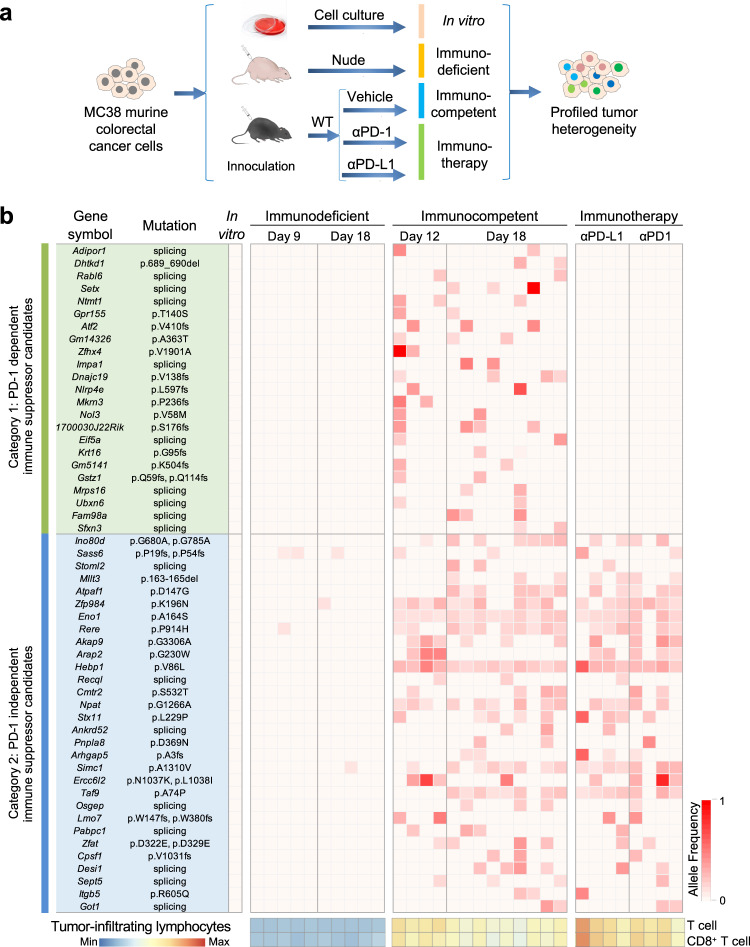
Clonal mutation profiling of tumors under progressive immune selection. **a** Schematic overview of clonal mutation profiling of MC38 cell line-derived tumors grown in host mice harboring different levels of T cell immunity. **b** Heatmap showing allele frequency (AF) of hotspot mutations enriched (AF > 0.1) in at least two different tumors from the immunocompetent group (*n* = 10 mice for immunodeficient, *n* = 13 for immunocompetent, and *n* = 8 for immunotherapy group). A total of 59 mutations in 53 genes were categorized to PD-1–dependent and –independent groups as indicated. Tumor-infiltrating lymphocytes were calculated by mMCP counter. See also Supplementary Fig. 1. Source data are provided as a source data file.

Cancer cell lines are genetically unstable and heterogeneous by natural selection^33,34^. We hypothesized that host immune pressure can selectively enrich cells expressing genes acquiring immune-escaping mutations during tumor expansion. To unravel such mutations, we implanted the MC38 murine colorectal cancer (CRC) cell line to C57BL/6 mice (wildtype, WT), and treated them with monoclonal antibodies against mouse programmed death protein 1 (PD-1) or programmed cell death ligand protein 1 (PD-L1). Nude mice were used as a control host for tumor growth under minimum T cell selective pressure (Supplementary Fig. 1a). Engrafted tumors were dissected and categorized as immunodeficient (*n* = 10), immunocompetent (*n* = 13), and immunotherapy groups (*n* = 8) for deep (40 M reads of) RNA sequencing (Fig. 1a).

Compared to the immunodeficient group, the expression signature of key immunomodulators and pathways of adaptive immunity^35^ were significantly enhanced in the immunocompetent group and further enhanced in the immunotherapy group, indicating escalating immune pressure (Supplementary Fig. 1b–d). After calling mutations using Genome Analysis Toolkit (GATK)^36^, we identified 59 non-synonymous somatic mutations (24 missense, 18 splicings, 15 frameshifts, 2 deletions) from expressed transcripts^34,37^ that were uniquely present in at least two different immunocompetent tumors, compared with cultured cells or any immunodeficient tumors. Among 53 genes affected by these mutations, 23 exhibited no alteration in any immunotherapy tumor, suggesting that cells with mutations in these genes might be eliminated by T cells after blockade of PD-1/PD-L1 checkpoint. The remaining 30 were found in tumors from both immunocompetent and immunotherapy groups, suggesting that they might facilitate cancer escape from T cell immunity independent of the PD-1 checkpoint (Fig. 1b).

### Targeted in vivo CRISPR screen for mutations driving immune evasion

**Fig. 2:**
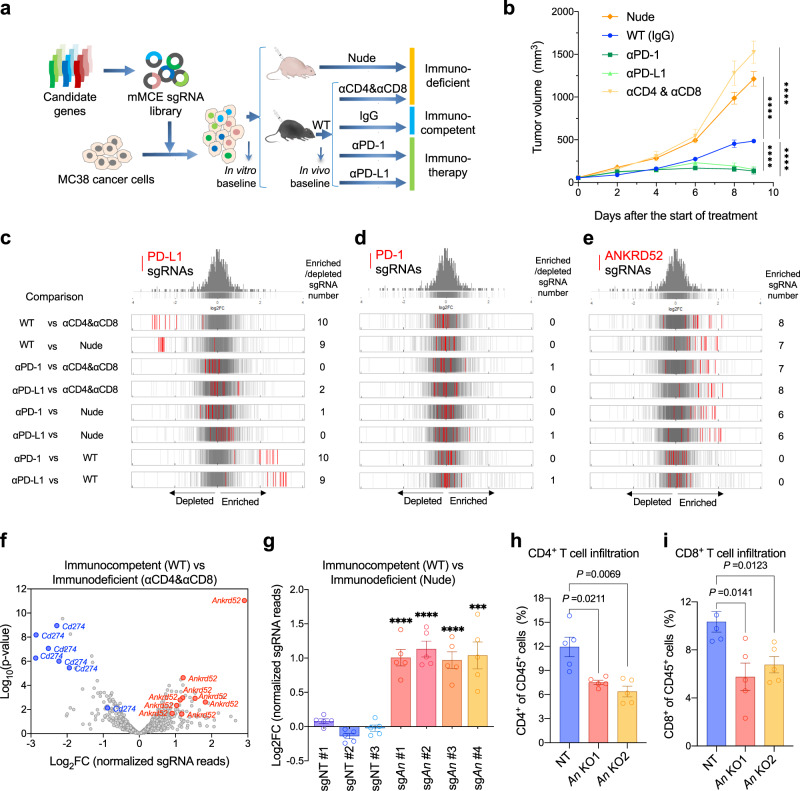
In vivo CRISPR screen targeting profiled mutations identifies ANKRD52 as a key modulator of cancer immunity. **a** Schematic overview of in vivo CRISPR screen to validate candidates from immune-selected mutations. **b** Tumor growth curves of MC38 tumors in nude mice (*n* = 8), and WT mice treated with rat IgG2a and IgG2b isotype (*n* = 8), PD-1 antibody (*n* = 10), PD-L1 antibody (*n* = 10), or CD4 and CD8 antibodies (*n* = 7). Data are represented as mean ± s.e.m., ****P < 0.0001, significance was determined using two-way analysis of variance (ANOVA). **c**–**e** Distribution histograms of log_2_ Fold-change (FC) for all 10 sgRNAs targeting *Cd274* (**c**), *Pdcd1* (**d**) or *Ankrd52* (**e**) as indicated in red lines, overlaid on gray gradient depicting the overall distribution (Cut-off: |FC | > 1.5, *P* < 0.05 for enrichment or depletion, analyzed by edgeR). **f**, Volcano plot for selected top guides for *Cd274* and *Ankrd52* (Cut-off: |FC | > 1.5, *P* < 0.05, analyzed by MAGeCK). **g** In vivo competition assay with equal number mixture of MC38 cells infected with sgRNA for non-targeting control (NT) or *Ankrd52* (*An*) in WT mice (*n* = 5) and nude mice (*n* = 5). Data are represented as mean ± s.e.m., ***P = 0.0004, ****P < 0.0001, significance was determined using two-tailed unpaired Student’s *t*-test to compare sg*Ankrd52* vs sgNT #2. **h**, **i**, Flow cytometry analysis of CD4^+^ (**h**) and CD8^+^ (**i**) T cell populations from NT and *Ankrd52* knockout (*An* KO) tumors (*n* = 5 per group). Data are representative of two independent experiments and represented as mean ± s.e.m., significance was determined using two-tailed unpaired Student’s *t*-test. See also Supplementary Fig. 2–5. Source data are provided as a source data file.

To validate the mutations that drive cancer evasion from T cells^8^, we created a library composed of single-guide (sg) RNAs targeting these 53 candidate genes (10 sgRNAs per gene) and positive control genes (*Pdcd1*, *Cd274*, *Jak1*, *Jak2,* and *B2m*), named as mouse Mutagenesis from Cancer Evolution, or mMCE, library (Supplementary Fig. 2a). This library was introduced to MC38 cells (>1000 × coverage) and implanted to T cell-depleted mice (Nude mice or CD4 and CD8 antibodies treatment), or WT mice with or without PD-1/PD-L1 antibody treatment (Fig. 2a). As expected, loss of T cells accelerated tumor growth, while blocking the PD-1 checkpoint inhibited tumor growth (Fig. 2b; Supplementary Fig. 2b). Tumors grown under these variable immune pressures were collected for amplicon sequencing to determine the sgRNA representation (Supplementary Fig. 2c), which revealed a very high consistency between biological replicate tumors in each group (Supplementary Fig. 2d; *Pearson*’s correlation (COR) > 0.58 for every two tumors).

MAGeCK and EdgeR analyses of guide abundance revealed that almost all PD-L1 sgRNAs were depleted in tumors from WT mice compared to T cell-depleted mice, but enriched in immunotherapy tumors compared to WT tumors (Fig. 2c; Supplementary Data 1), consistent with the inhibitory function of PD-L1 in cancer cells to induce exhaustion of PD-1-expressing T cells^38^. The same distribution was observed for sgRNAs targeting *Jak1* and *Jak2* (Supplementary Fig. 3a–d; Supplementary Data 1), which mediate the response to anti-PD-1 therapy in melanoma patients^39^. Similar to our results, sgRNAs for key IFNγ signaling genes including *Jak1/2* have been reported to exhibit different or even opposing performance between in-vivo and in-vitro CRISPR library screens^10,40^. Inhibiting IFNγ signaling in low/absent MHC-I tumors stimulated the production of IFNγ by exhausted T cells, which drove the maturation of innate immune cells to kill tumor cells^41^. By contrast, sgRNAs targeting PD-1, mainly expressed on activated T cells but not on tumor cells^42^, did not exhibit obvious abundance changes in tumors across all groups (Fig. 2d). These results corroborate the screen robustness.

### Resistance to T cell immunity by ANKRD52 loss

Furthermore, we found that 6–8 out of 10 sgRNAs targeting *Ankrd52* were significantly enriched in WT and immunotherapy tumors compared to T cell-depleted tumors (Fig. 2e, f), suggesting that inactivation of ANKRD52 conferred a selective advantage for tumor cells against PD-1 independent T cell-mediated immunity. The significance of this distribution pattern is reinforced by the presumably loss-of-function splice-site alteration (NM_172790.2:c.2723-2 A > G) in *Ankrd52* that was enriched in both WT and immunotherapy tumors (Fig. 1b). Although alterations of several genes (such as *Hebp1*, *Eno1*, and *Taf9*) other than *Ankrd52* are more frequently detected in our tumor profiling, their targeting sgRNAs exhibited much less enrichment or fold-change as compared to those of *Ankrd52* (Supplementary Fig. 3e; Supplementary Data 1). We, therefore, selected *Ankrd52* for further studies.

To validate the role of *Ankrd52* in immune evasion, we first performed in vivo competition assays by infecting MC38 cells individually with four *Ankrd52* sgRNAs and three non-target sgRNAs, followed by implanting the mixture of the same number of cells from the 7 pools to WT or nude mice (Supplementary Fig. 4a, b). As expected, each *Ankrd52* guide became significantly enriched in WT tumors compared to nude tumors, indicating growth advantages over control cells (Fig. 2g). Next, we generated *Ankrd52-*null MC38 cells using CRISPR-Cas9 gene editing and found these cells exhibited growth disadvantage over extended time in long-term culture or as tumors grown in T cell-depleted mice (Supplementary Fig. 4c, d), a phenomenon supported by CRISPR library screen results (Supplementary Fig. 4e, f) and likely as a result of p53/p21 activation (Supplementary Fig. 4g). However, the growth disadvantage of *Ankrd52-*null tumors was abolished when implanted to WT mice (Supplementary Fig. 5a), suggesting that loss of ANKRD52 enabled cancer cells to escape T cell immunity in vivo, thereby offsetting their intrinsic growth deficiency. Tumor-infiltrating lymphocyte (TIL) analysis revealed that the proportions, but not activation or cytotoxicity, of CD4^+^ and CD8^+^ T cells were significantly decreased in *Ankrd52-*null tumors (Fig. 2h, I; Supplementary Fig. 5b–g). Exhausted T cells (LAG3^+^ PD1^+^ or TIM3^+^ PD1^+^) were also decreased (Supplementary Fig. 5h, i), possibly explaining why *Ankrd52* mutation was not eliminated by PD-1 blockade in our tumor mutation profiling (Fig. 1b).

### ANKRD52 is a direct modulator for T cell killing

**Fig. 3:**
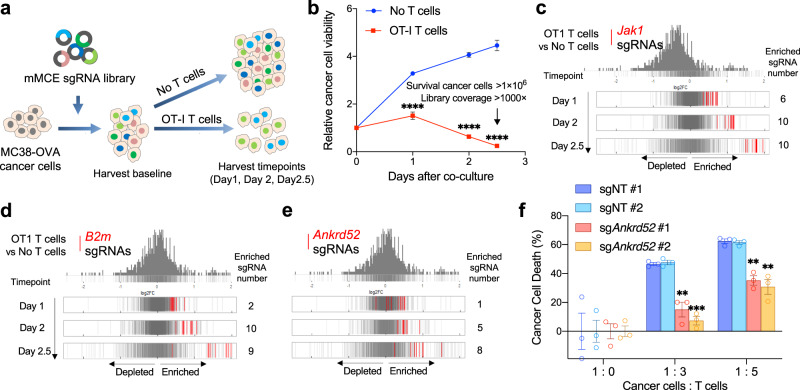
Targeted in vitro CRISPR screen identifies ANKRD52 as a direct modulator of cancer sensitivity to T cell cytotoxicity. **a** Schematic overview of CRISPR screen using co-culture of OT-I T cell with MC38-OVA cells. **b** Cell viability of MC38-OVA cells co-cultured with OT-I T cells during 2.5 days (*n* = 3 per group per timepoint). Data are represented as mean ± s.d., ****P < 0.0001, significance is determined using multiple two-tailed Student’s *t*-test. **c**–**e** Distribution histograms of log_2_FC values for all ten sgRNAs targeting *Jak1* (**c**), *B2m* (**d**), or *Ankrd52* (**e**) (Cut-off: FC > 1.4, *P* < 0.05 for enrichment, analyzed by edgeR). **f** Killing of MC38-OVA cells with indicated sgRNAs by OT-I T cells at indicated ratio in co-culture. Data are representative of three independent experiments and represented as mean ± s.e.m., **P < 0.01, ***P < 0.001, significance was determined using multiple two-tailed Student’s *t*-test (sgNT #1 vs sg*Ankrd52* #1, *P* = 3.72×10^−3^ for 1:3, *P* = 1.74×10^−3^ for 1:5; sgNT #2 vs sg*Ankrd52* #1, *P* = 3.64×10^−3^ for 1:3, *P* = 1.67×10^−3^ for 1:5; sgNT #1 vs sg*Ankrd52* #2, *P* = 2.28 × 10^−4^ for 1:3, *P* = 4.43 × 10^−3^ for 1:5; sgNT #2 vs sg*Ankrd52* #2, *P* = 2.7 × 10^−4^ for 1:3, *P* = 4.57 × 10^−3^ for 1:5). See also Supplementary Figs. 6 and 7. Source data are provided as a source data file.

To further investigate whether ANKRD52 or other immune evasion mutations participate in cancer sensitivity to T cell-mediated cytotoxicity directly, we performed a parallel in vitro CRISPR screen whereas MC38 cells expressing ovalbumin (MC38-OVA) were introduced with the mMCE sgRNA library and then co-cultured with antigen-specific OT-I T cells (Fig. 3a, b; Supplementary Fig. 6a, b). Like *Jak1/2* and *B2m*, *Ankrd52* sgRNAs exhibited time-dependent enrichment in tumor cells in the presence of T cells (Fig. 3c–e; Supplementary Fig. 6c–g; Supplementary Data 2), providing direct evidence that loss of ANKRD52 conferred resistance to T cell killing. MC38-OVA or OVA peptide-treated MC38 cells with depleted ANKRD52 became less sensitive to the killing by OT-I T cells and suppressed the proliferation of T cells when co-cultured (Fig. 3f; Supplementary Fig. 7). Putting together, CRISPR library screens in vitro and in vivo demonstrate a direct role of ANKRD52 in modulating cancer sensitivity to T cell-mediated clearance.

### Attenuated IFNγ response by ANKRD52 loss

**Fig. 4:**
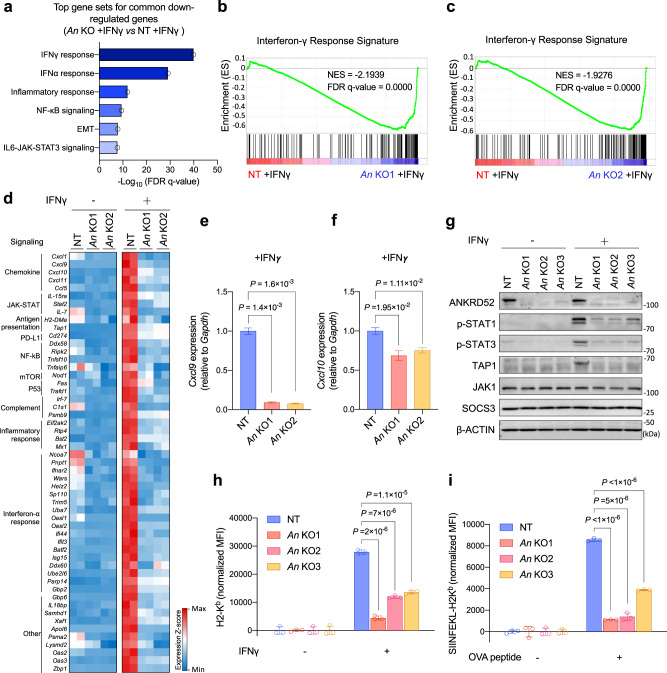
ANKRD52 inactivation attenuates IFNγ signaling, chemokine expression, and antigen presentation. **a** Hallmark gene sets enriched for commonly down-regulated genes in *Ankrd52*-null (both *An* KO1 and *An* KO2, 1.5-fold-change cut-off, *P* < 0.05, analyzed by edgeR) MC38 cells compared to control cells after IFNγ treatment. **b**, **c** Enrichment of genes associated with IFNγ response in *Ankrd52*-null cells exposed to IFNγ. **d** Heatmap showing down-regulated IFNγ responsive gene expression in *Ankrd52*-null cells by RNA-seq analysis (*n* = 2 per group per condition). **e**, **f** *Cxcl9* (**e**) and *Cxcl10* (**f**) mRNA level in control and *ANKRD52*-null MC38 cells treated with IFNγ (*n* = 3 per group). Data are representative of two independent experiments and represented as mean ± s.e.m., significance was determined using two-tailed unpaired Student’s *t*-test. **g** Abundance of IFNγ signaling proteins in control and *Ankrd52*-null MC38 cells treated with IFNγ. Data are representative of five independent experiments. **h** Abundance of membrane MHC-I expression in control and *Ankrd52*-null MC38 cells after treatment with IFNγ. MFI (mean fluorescence intensity) of H2-K^b^ was normalized by the responding group without IFNγ treatment (*n* = 3 per group per condition). Data are representative of four independent experiments and represented as mean ± s.d., significance was determined using two-tailed unpaired Student’s *t*-test. **i** Presentation of OVA-derived peptide (SIINFEKL) in OVA-treated control and *Ankrd52*-null MC38 cells. MFI of SIINFEKL-H2K^b^ was normalized by the responding group without IFNγ treatment (*n* = 3 per group per condition). Data are representative of three independent experiments and represented as mean ± s.d., significance was determined using two-tailed unpaired Student’s *t*-test. See also Supplementary Figs. 8 and 9. Source data are provided as a source data file.

We next sought to determine how ANKRD52 alters cancer cell’s interaction with T cell by comparing the transcriptome of *Ankrd52-*null cells with control cells. Loss of ANKRD52 caused a remarkable downregulation in the expression of IFNγ and IFNα responsive gene sets after MC38 cells were stimulated with IFNγ (Fig. 4a–d; Supplementary Fig. 8a–d; Supplementary Data 3), a T cell cytokine essential for tumor immunity^43^. Among them were PD-L1 and effector T cell-recruiting chemokines such as CXCL9 and CXCL10 (Fig. 4d)^44^, an observation confirmed by flow cytometry analysis and quantitative PCR (Fig. 4e, f; Supplementary Fig. 9a–c) and in accordance with the decreased infiltration of T cells in *Ankrd52-*null tumors (Fig. 2h, i). Strikingly, IFNγ-induced expression of TAP1, a key component of the antigen processing machinery, was completely abrogated by ANKRD52 depletion (Fig. 4d, g). Accordingly, significant decrease in total surface MHC-I was observed in both *Ankrd52-*null MC38 cancer cells isolated from tumors (Supplementary Fig. 9d, e) or cultured with IFNγ stimulation (Fig. 4h; Supplementary Fig. 9f). Presentation of the SIINFEKL epitope from OVA in the context of H2K^b^ was also significantly lower in IFNγ-stimulated ANKRD52-deficient cells than control cells (Fig. 4i; Supplementary Fig. 9g, h), suggesting that loss of ANKRD52 decreased the levels of antigen-loaded MHC-I on the surface of tumor cells and thus compromised their recognition by T cells.

### Impaired IFNγ response by clinical *ANKRD52* mutations

**Fig. 5:**
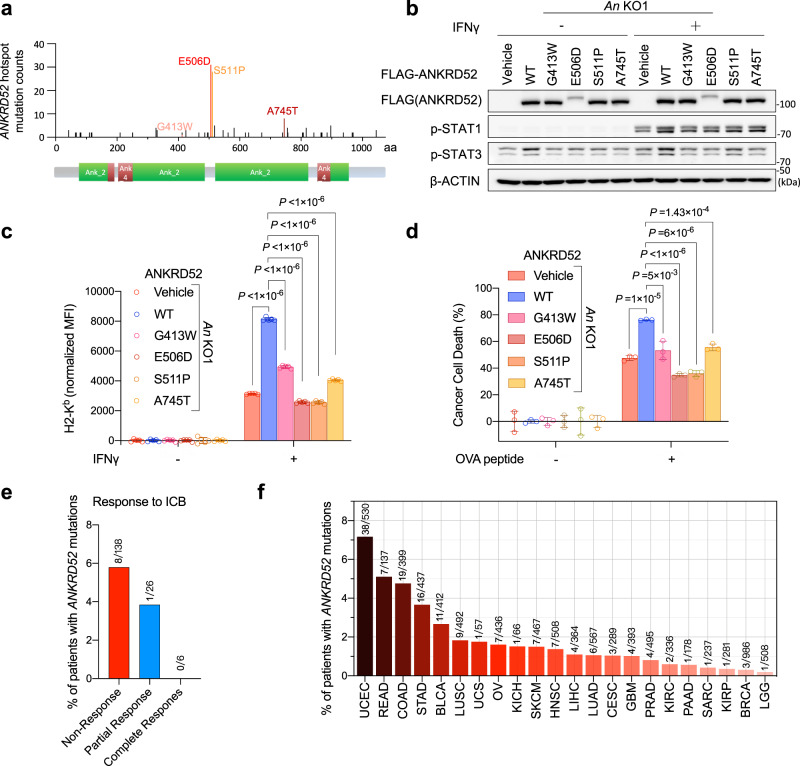
Hotspot ANKRD52 mutations in patients impair IFNγ and T cell response. **a** Localization of clinical hotspot *ANKRD52* mutations from combined COSMIC and OncoWuXi database. **b** Protein abundance of p-STAT1 and p-STAT3 in *Ankrd52*-null MC38 cells expressing WT or indicated mutant ANKRD52 after IFNγ treatment. Data are representative of two independent experiments. **c** Membrane MHC-I level in *Ankrd52*-null MC38 cells expressing WT or indicated mutant ANKRD52 after IFNγ treatment. MFI of H2-K^b^ was normalized by the responding group without IFNγ treatment (*n* = 5 per group per condition). Data are representative of two independent experiments and represented as mean ± s.d., significance was determined using two-tailed unpaired Student’s *t*-test. **d** Killing of OVA-treated *Ankrd52*-null MC38 cells expressing WT or mutant ANKRD52 by OT-I T cells (*n* = 3 per group per condition). Data are representative of two independent experiments and represented as mean ± s.d., significance was determined using two-tailed unpaired Student’s *t*-test. **e** Bar plot showing the percentage of patients with mutations in *ANKRD52* across cancer patients receiving ICB therapies (anti-CTLA-4 or anti-PD-1) reported by Van Allen et al., *Science* 2015 and Riaz et al., *Cell* 2017. The non-response group is composed of SD and PD. **f** Bar plot showing the percentage of patients with mutations in *ANKRD52* across multiple cancer types. Source data are provided as a source data file.

IFNγ response is regulated by JAK-STAT signaling^45^. Activation of STAT1 by IFNγ was markedly diminished by ANKRD52 depletion (Fig. 4g). Compared with the wildtype gene, four hotspots *ANRKD52* mutations (G413W, E506D, S511P and A745T) identified in cancer patient samples (Fig. 5a) failed to effectively rescue the activation defect of STAT1 (Fig. 5b) or upregulation of membrane MHC-I levels (Fig. 5c) upon IFNγ treatment, and thus maintained cancer cell resistance to T cell-mediated killing (Fig. 5d). Analysis of several published datasets on cancer patients receiving immunotherapies^46,47^ revealed a higher *ANKRD52* mutation rate in tumor samples from non-responders than responders (Fig. 5e). Given a 4.8% prevalence of *ANKRD52* mutations (19/399) in colorectal adenocarcinoma and 1.5% (153/10182) in all cancer types (Fig. 5f), our studies suggest that these patients might not benefit as much from T cell-based immunotherapies.

### Silencing of *SOCS1* by ANKRD52-regulated miRNA targeting

**Fig. 6:**
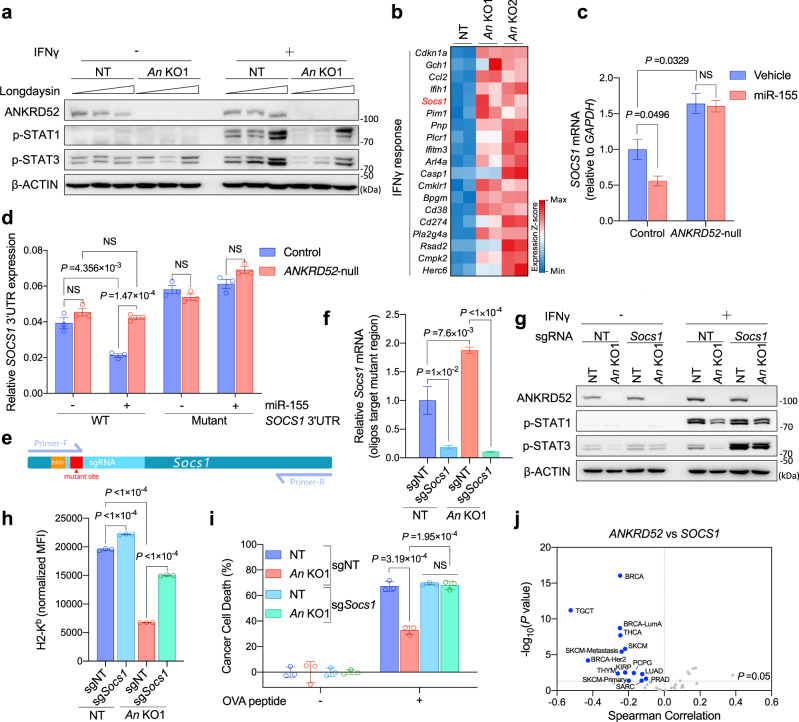
ANKRD52 is required for miRNA-mediated silencing of SOCS1 to regulate T cell response. **a** p-STAT1 and p-STAT3 abundance in MC38 cells treated with IFNγ and increasing Longdaysin (0, 50, 100 μM, a CK1α inhibitor). Data are representative of three independent experiments. **b** Heatmap showing commonly upregulated gene expression of IFNγ signaling by RNA-seq analysis of *Ankrd52*-null MC38 cells (*n* = 2 replicates per group). **c** *SOCS1* mRNA level in control and *ANKRD52*-null 293 T cells overexpressing miR-155 (*n* = 3 per group per condition). Data are representative of two independent experiments and represented as mean ± s.e.m., significance was determined using two-tailed unpaired Student’s *t*-test. **d** Activity of WT and mutant *SOCS1* 3’UTR (Rluc/Fluc) in a dual-luciferase reporter in 293 T cells overexpressing miR-155 (*n* = 3 per group per condition). Data are representative of three independent experiments and represented as mean ± s.e.m., significance was determined using two-tailed unpaired Student’s *t*-test. **e** Schematic overview of qPCR test targeting mutant region to check *Socs1* knockout efficiency. **f** *Socs1* mRNA level tested by qPCR targeting mutant region in *Ankrd52*-null MC38 cells with inactivated SOCS1 (*n* = 5 per group). Data are represented as mean ± s.e.m., significance was determined using two-tailed unpaired Student’s *t*-test. **g** p-STAT1 and p-STAT3 abundance in *Ankrd52*-null MC38 cells with inactivated SOCS1 after IFNγ treatment. Data are representative of three independent experiments. **h** Membrane MHC-1 expression in *Ankrd52*-null MC38 cells with inactivated SOCS1 after IFNγ treatment. MFI of H2-K^b^ was normalized by the responding group without IFNγ treatment (*n* = 3 per group per condition). Data are representative of two independent experiments and represented as mean ± s.d., significance was determined using two-tailed unpaired Student’s *t*-test. **i** Killing of OVA-treated *Ankrd52*-null MC38 cells with inactivated SOCS1 by OT-I T cells (*n* = 3 per group per condition). Data are representative of two independent experiments and represented as mean ± s.d., significance was determined using two-tailed unpaired Student’s *t*-test. **j** Volcano plot showing the Spearman’s correlation and estimated significance of *ANKRD52* with *SOCS1* mRNA levels across all TCGA cancer types. Each dot represents a cancer type, blue dots indicate significant negative correlations (*P* < 0.05, TIMER). See also Supplementary Figs. 10 and 11. Source data are provided as a source data file.

ANKRD52, an Ankyrin repeat protein, forms a complex with the PPP6C phosphatase to dephosphorylate Argonaute 2 (AGO2) to promote miRNA-mediated gene silencing, a process counteracted by CK1α-induced phosphorylation of AGO2^21^. Blocking CK1α rescued the impaired phosphorylation of STAT1 in IFNγ-stimulated ANKRD52-deficient cells (Fig. 6a; Supplementary Fig. 10a), implicating a potential role of the AGO2 phosphorylation cycle in regulating IFNγ response.

We, therefore, hypothesized that certain miRNA-silenced targets were upregulated upon ANKRD52 deletion to suppress the IFNγ response. Gene set enrichment analysis (GSEA) of MC38 transcriptome identified 19 IFNγ signaling genes significantly upregulated in ANKRD52-deficient cells compared to control cells (Fig. 6b; Supplementary Fig. 10b; Supplementary Data 3), among which SOCS1 was also shown to be similarly controlled by ANKRD52 in human colorectal carcinoma HCT116 cells^21^ (Supplementary Fig. 10c). SOCS1 suppresses JAK-STAT signaling and cancer immunotherapy^11,45^, and its mRNA levels were reciprocally controlled by ANKRD52 and CK1α (Supplementary Fig. 10d, e). Overexpression of miR-155, the major *SOCS1*-targeting miRNA^48^, in 293 T cells led to ANKRD52-dependent repression of *SOCS1* mRNA levels (Fig. 6c; Supplementary Fig. 10f). Using a luciferase reporter fused to the *SOCS1* 3′UTR harboring an intact or mutant miR-155 seed binding sequence^48^, we found that miR-155-mediated *SOCS1* silencing required both ANKRD52 expression and correct miRNA targeting (Fig. 6d). CRISPR-mediated disruption of miR-155 in MC38 cells resulted in an increase of *Socs1* mRNA level (Supplementary Fig. 11a), and a correspondent decrease of IFNγ-stimulated responses and T cell-mediated cytotoxicity (Supplementary Fig. 11b–g). Importantly, deletion of SOCS1 in *Ankrd52*-null MC38 cells (Fig. 6e, f) rescued the impaired STAT1 phosphorylation, the decreased membrane MHC-I level, and the dampened T cell-mediated cytotoxicity (Fig. 6g–i). Notably, TIMER analysis of the TCGA datasets^49^ revealed a strong negative correlation between transcript levels of *ANKRD52* and *SOCS*1 in many human cancer types (Fig. 6j). Taken together, these results indicate that ANKRD52 regulates cancer immunity mainly through miR-155-mediated silencing of *SOCS1*.

### Regulation of cancer immunity by miRNA machinery

**Fig. 7:**
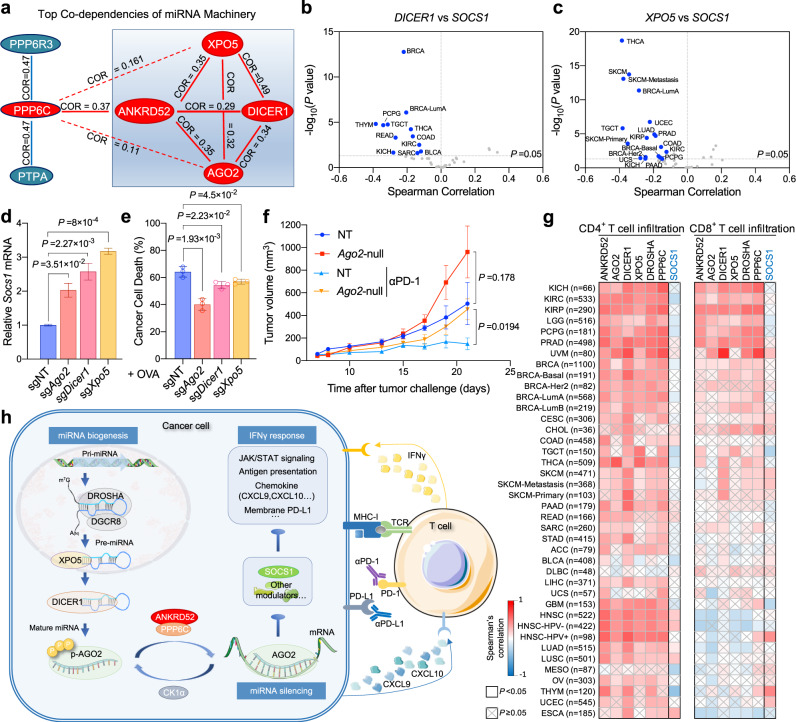
Inactivation of miRNA machinery promotes cancer-intrinsic escape from T cell elimination. **a** Diagram showing the Spearman’s correlation of top co-dependent proteins with ANKRD52 or PPP6C in CRISPR (Avana) Public 20Q3 database. Solid lines depict significant positive correlations (Correlation > 0.25, *P* < 0.001) and dashed lines depict weak correlation (Correlation > 0.1, *P* < 0.01). **b**, **c** Volcano plot showing the Spearman’s correlation and estimated significance of *DICER1* (**b**) or *XPO5* (**c**) with *SOCS1* mRNA levels from RNA-seq data across all TCGA cancer types. Each dot represents a cancer type in TCGA; blue dots indicate significant negative correlations (*P* < 0.05, TIMER). **d** *SOCS1* mRNA level in MC38 cells with targeted sgRNAs (*n* = 3 per group). Data are representative of three independent experiments and represented as mean ± s.e.m., significance was determined using two-tailed unpaired Student’s *t*-test. **e** Killing of OVA-treated MC38 cells with targeted sgRNAs by OT-I T cells (*n* = 3 per group per condition). Data are representative of two independent experiments and represented as mean ± s.d., significance was determined using two-tailed unpaired Student’s *t*-test. **f** Tumor growth curves of *Ago2*-null or control MC38 tumors in WT mice treated with PD-1 antibody or not (*n* = 5 for NT tumor, *n* = 6 for NT with anti-PD-1 and *n* = 7 for *Ago2*-null with or without anti-PD-1). Data are represented as mean ± s.e.m., significance was determined using two-tailed unpaired Student’s *t*-test. **g** Heatmap showing the Spearman’s correlation of *ANKRD52*, *AGO2*, *DICER1*, *XPO5*, *DROSHA*, *PPP6C*, or *SOCS1* mRNA levels with CD4^+^ and CD8^+^ T cell abundance in tumors across all TCGA cancer types. **h** Model of miRNA machinery in regulation of cancer-intrinsic evasion from T cell attack. See also Supplementary Figs. 12–15. Source data are provided as a source data file.

Before targeted to complementary mRNA, mature miRNA is produced from precursor miRNA, which is cleaved from primary miRNA by the DROSHA-DGCR8 complex, exported from the nucleus to the cytoplasm by XPO5, and finally processed by DICER1^20^. Analysis of 789 genome-wide CRISPR screen data (CRISPR Avana Public 20Q3, depmap.org) revealed a strong positive co-dependency between ANKRD52 and any of the core miRNA biogenesis and targeting machinery (Fig. 7a; Supplementary Fig. 12a). Like *ANKRD52*, transcript levels of these core miRNA processors negatively correlate with the *SOCS1* level in a large number of human cancer types (Fig. 7b, c; Supplementary Fig. 12b–e). Consistently, CRISPR-mediated inactivation of *Ago2*, *Dicer1*, *Xpo5*, and *Dgcr8* in MC38 cells increased *Socs1* mRNA (Fig. 7d), and reduced downstream IFNγ-stimulated responses (STAT1 activation and antigen presentation), as well as mitigated T cell-mediated killing (Fig. 7e; Supplementary Fig. 12f–j, 13a–g, 14a–g). Moreover, MC38 tumors with inactivated AGO2 exhibited growth advantage in the immunocompetent host with increased T cell pressure induced by PD-1 blockade treatment (Fig. 7f; Supplementary Fig. 14h). Remarkably, analysis of TCGA datasets revealed that expression of these miRNA biogenesis (*DICER1*, *XPO5,* and *DROSHA*) and targeting (*AGO2*, *PPP6C,* and *ANKRD52*) machinery components all positively correlated with the intratumoral T cell abundance across almost all human cancer types, while *SOCS1* expression did not exhibit such correlation (Fig. 7g).

Additionally, mutations in the miRNA machinery are found in 4–7% CRC patient samples (Supplementary Fig. 15a). Some of these mutations were associated with worse survival of CRC patients exhibiting a higher degree of cytotoxic T lymphocyte infiltration (Supplementary Fig. 15b). In gene expression–based consensus molecular subtyping (CMS), a robust CRC classification system^50^, miRNA machinery mutations were enriched in CMS1 (Supplementary Fig. 15c), a subtype linked to strong immune activation, poor patient survival after relapse, and bleak prognosis in metastatic CRC^50,51^. Therefore, inactivation of the miRNA machinery not only protects tumor cells from T cell-mediated cytotoxicity but also results in a pro-survival tumor microenvironment (Fig. 7h).

## Discussion

Cancer cells accumulate a myriad of genetic and epigenetic alterations to enable escaping from host immune elimination^33^. It is a great challenge to single out those alterations driving immune evasion and to unravel how they empower cancer cells against hostile immunity^7^. Here, we combined immune selection and CRISPR screen validation in vivo to identify spontaneous or rare mutations in cancer cells that endow resistance to T cell-mediated cytotoxicity. Many of the mutations affected single residue replacement (24/59; Fig. 1b) and may result in the gain of function in the corresponding proteins, which will not be well represented in our conventional CRISPR knockout screen. The interaction between these mutations and host immunity can be further explored by editing the cancer cell genome individually or in batch with the CRISPR base-editing tools^52^. Regardless, the same tumor profiling and CRISPR screening approach can be applied to different cancer cell lines and variable immune selection to untangle the cancer genetic heterogeneity, for identification of alterations that enable cancer cells to evade innate and adaptive immunity, and locating additional immunotherapeutic strategies from them. For example, we can profile mutations in syngeneic tumors from mice with or without depletion of NK cells or macrophages and interrogate genes critical for cancer cells to survive these innate immune cells. Alternatively, we can randomly mutagenize mouse cancer cell lines to increase the odds of mutant clone expansion upon selection from host immunity or immunotherapy during tumor growth or metastasis.

Loss of function mutation in *Ankrd52* was enriched in cell line-derived tumors grown specifically under T cell-mediated immune pressure (Fig. 1b). ANKRD52 forms a complex with PPP6C to counteract CK1α-mediated AGO2 phosphorylation and promote miRNA loading to target mRNA^21^. Like *ANKRD52*, which is mutated in 4.8% colorectal adenocarcinoma and 1.5% of all cancer types (Fig. 5f), *PPP6C* harbors recurrent and potentially driver mutations, such as the R264C mutation in 3% melanoma, which may disrupt PPP6C binding to ANKRD52 and thus the enzymatic activity^28^. Consistently, keratinocyte-specific deletion of *Ppp6c* promotes tumor formation in mice^53^. The other core miRNA pathway genes are also frequently mutated in cancer. *DROSHA* is frequently mutated in Wilms tumor samples with more than 70% mutations occurring at a metal-binding residue E1147 in the RNase III domain^26,27^. DICER1 is considered a haploinsufficient tumor suppressor, as loss of a single *Dicer1* allele promotes tumorigenesis and reduces survival in mice^29,54^. Its mutations are frequently found in different cancer types and contribute to metastasis and poor survival in patients^20,23–25,30^. *XPO5*‑inactivating mutations are detected in endometrial, colon, gastric, and breast tumors with microsatellite instability, causing impaired pre-miRNA export and are associated with increased cancer risk^55,56^.

Here, we show that the abundant mutations in these core miRNA machinery components may not only promote uncontrolled cancer cell-intrinsic proliferation but also enable them to resist T cell-mediated elimination and thus confer an additional survival advantage to cancer cells in a hostile immune environment. Inactivation of the machinery down-regulates T cell recruitment chemokines CXCL9 and CXCL10 (Fig. 4d–f), and remodels a more favorable microenvironment for tumor growth (Fig. 2h, i). In most human cancers, expression of the core machinery components exhibits very strong positive correlations with tumor-intrinsic T cell infiltrations (Fig. 7g). Notably, CXCL9 expression is regarded as a strong predictor of ICB response in a meta-analysis of over 1000 ICB-treated patients across seven tumor types^57^. Our results suggest that a sizeable patient subpopulation with defective miRNA activity may not respond well to T cell-based immunotherapies.

Despite the recent remarkable clinical success of PD-1/PD-L1 ICB, most cancer patients fail to respond or develop treatment resistance, frequently due to defective antigen presentation and IFNγ signaling^1,4,13,14^. We demonstrate that the core miRNA biogenesis and targeting machinery are essential for the IFNγ-activated JAK-STAT signaling and antigen presentation in cancer cells, largely by controlling miR-155-targeted silencing of *SOCS1* (Fig. 7h). Unlike our observation in CRC cells, *Dicer* inactivation was reported to result in increased IFN signaling via Let-7 miRNA in macrophages and embryonic stem cells^58,59^, both primary cells. This discrepancy may be due to the heterogeneous mutations in cancer cells that dictate distinct IFN response upon *Dicer* inactivation. IFNγ signaling is regulated by multiple positive and negative feedbacks, thus playing different or even opposing roles in cancer cells and immune cells^41,60,61^. Interestingly, depletion of SOCS1 was also found to sensitize cancer cells to PD-1 blockade in the previous in vivo CRISPR library screen^11^. Although expression levels of individual miRNA machinery components are inversely correlated with SOCS1 in many human cancer types (Fig. 6j; Fig. 7b, c), inactivation of the core miRNA pathway should block silencing of many more genes in addition to *SOCS1* for immune response, which still need to be further elucidated. Additionally, we showed that chemical inhibition of the CK1α kinase activity can counteract the decreased IFNγ response (Fig. 6a). CK1α could also be degraded by lenalidomide^62^, a clinically proved anti-myeloma drug reported to enhance T cell-mediated anti-tumor activity and ICB response^63^. Our data suggest that targeting the miRNA machinery in combination with immunotherapies may provide a cancer therapeutic strategy.

## Methods

### Animals

The protocol and any amendment(s) or procedures involving the care and use of animals in this study were in accordance with Shanghai Institutional Animal Care and Use Committee (IACUC) guidelines and approved by IACUC of ShanghaiTech University or WuXi AppTec. Animal experiments were conducted at the National Facility for Protein Science or OIU of WuXi AppTec. C57BL/6 J and *Foxn1*^nu^ (nude) mice (female, aged at 6–8 weeks and weighing approximately 18–22 g) were used for tumor implantation experiments and purchased from Shanghai SLAC Laboratory Animal. Mice were housed at approximately 20–26 °C, 40–70% humidity on a 12-hr light/dark cycle in a special pathogen-free environment and in individual ventilation cages (four mice per cage). Tumor burden did not exceed 10% of the animal’s body weight for each mouse.

### Cell lines and cell culture

All cell lines used in this study were tested as mycoplasma-negative using the Universal Mycoplasma Detection Kit (ATCC, 30-1012 K). 293FT (R70007) and 293 T/17 (CRL-11268) cell lines were originally purchased from Thermo Fisher and ATCC, respectively, and cultured according to the manufacturers’ manual specifically. MC38 cell line was provided by WuXi AppTec and cultured using DMEM (Gibco) with 10% fetal bovine serum (FBS), respectively.

MC38-OVA cell lines were constructed by introducing cDNA of OVA peptide into the plenti6.3 vector (Thermo Fisher, K533000). The cDNA sequences for OVA peptide (p.257–264, SIINFEKL) were synthesized at GENEWIZ. Blasticidin (Gibco, A1113903) selection was used for lentivirus transduced cells (MOI ≈ 0.3) to generate OVA stably expressing cells.

OT-I T cells were isolated from the spleen and lymph nodes of OT-I transgenic mouse (a gift from Bing Sun’s Lab at SIBCB) by EasySep™ Mouse CD8^+^ T Cell Isolation Kit (STEMCELL, 19753). Fresh isolated CD8^+^ T cells were cultured in a complete T cell medium (RPMI-1640 with 10%FBS, 20 mM HEPES, 1 mM sodium pyruvate, 0.05 mM 2-mercaptoethanol, 2 mM L-glutamine, 50 U/ml streptomycin and penicillin), and treated with 2 ng/mL recombinant mouse IL-2 (Novoprotein, CK24).

### In vivo tumorigenesis under different immune selection

Murine tumor syngeneic model under different immune selective pressure was built using nude or C57BL/6 J mice with or without different antibody or IgG treatment. Each mouse was inoculated subcutaneously at the right flank with MC38 tumor cells (1 × 10^6^/mouse) in 0.1 mL of PBS for tumor development. Tumor measurement was taken manually by collecting the longest dimension (length, *L*) and the longest perpendicular dimension (width, *W*). Tumor volume was estimated with the formula: (*L* × *W*^2^)/2. When the average tumor volume reached approximately 40–60 mm^3^, the animals were performed with stratified randomization empirically based upon their tumor volumes and treatment was started. The test article administration for groups in all experiments of this research are shown in Table 1.

**Table 1:** Conditions for in vivo tumorigenesis.

Group	Mouse Strain	Treatment	Dose	Dosing volume	Dosing route	Schedule
Immunocompetent	C57BL/6 J	Rat IgG2a/b	2.5 mpk for each IgG	10 μL/g	i.p.	Every other day
Immunotherapy	C57BL/6 J	Anti-PD-1	5 mpk	10 μL/g	i.p.	Every other day
Immunotherapy	C57BL/6 J	Anti-PD-L1	5 mpk	10 μL/g	i.p.	Every other day
Immunodeficient	C57BL/6 J	Anti-CD4/CD8	500 μg	10 μL/g	i.p.	Every week
Immunodeficient	Nude	Rat IgG2a/b	2.5 mpk	10 μL/g	i.p.	Every other day

All the antibodies for mice injection were purchased from Bio X Cell and listed as follows: anti-PD-1 (BE0146), anti-PD-L1 (BE0101), anti-CD4 (BE0119), anti-CD8 (BE0117), IgG2a (BE0089), and IgG2b (BE0090). The study was terminated for tumor collection before the mean tumor volume of any immunodeficient group reached a value of 2000 mm^3^.

### RNA-Seq analysis

For tumors, collected tumors were cut large into samples with size ≤ 0.5 cm in dimension and then the fresh tissues were placed in 5–10 volumes of RNAlater® Solution (Thermo Fisher, AM7020). For cultured cells, *Ankrd52*-null or control MC38 cells were seeded onto 6-well plates at 5 × 10^5^ cells/well in duplicate and challenged with 10 ng/ml IFNγ (CST, 39127) for 24 h, with cytokine-free medium serving as control. RNA isolation, library construction, and sequencing were performed by Mingma Technologies. RNeasy MinElute Cleanup Kit (QIAGEN, 74204) was used for RNA extraction, followed by generating mRNA-focused sequencing libraries from total RNA using Illumina TruSeq RNA Sample Preparation Kit v2. Paired-end 150 bp sequencing was done on illumina Hiseq X10 (40 M reads for each sample). FastQC (0.11.2) was used to generate QC report and the adapters of each sample were trimmed, respectively.

To measure gene expression level, fastq file of each sample was mapped to the reference genome to get reads count table as input for differential expression analysis. The clean reads were mapped to the mouse (*Mus musculus*) genome (mm10) using STAR^64^ (2.4.2a) and annotated with a transcriptome database (gene code vM13). Gene abundance estimation by read counts was conducted with the software RSEM^65^ (1.2.29). Normalized tags per million (TPM) were calculated on the number of clean reads mapped to a specific region of the genome using the relative log expression (RLE) method in edgeR^66^ (3.16.5). Differentially expressed genes (DEG) refer to compared gene expression levels between two samples or two groups. DEG were defined by using the criterion that fold-change > 1.5 and adjust *P* < 0.05 (adjust *P*-value). Pathway enrichment analysis (GO and KEGG) on DEGs was performed on differentially expressed genes using GOstats^67^ (2.40.0) in R, with the threshold that *P* < 0.01. Gene set enrichment analysis for molecular signature^68^ was carried out using GSEA^69^ software with threshold that *P* < 0.01. Tumor-infiltrating lymphocytes were analyzed by mMCP counter^70^.

### Identification of mutations from profiled cancer heterogeneity

To detect mutations (SNP and InDel) in RNA-seq datasets from tumors under different immune selection, we used the aligner STAR (2.5.2b) to map the reads to the genome (GRCm38 - mm10) and used MarkDuplicates in picard-tools-1.94 (http://broadinstitute.github.io/picard) to dedup the bam file^64^. And then, we applied a joint call to detect SNP and Indels across all RNA-seq datasets by following the GATK^36^ (3.7.0) best-practice guidelines (https://gatk.broadinstitute.org/hc/en-us/sections/360007226651-Best-Practices-Workflows). In the variant filtering step, we used aspecific hard filter to get the filtered VCF file. Then, we used ANNOVAR^71^ to annotate the mutation sites. Finally, we only kept the mutations in exonic and splicing regions for further study. Mutation allele frequency was counted with the formula: depth of the alteration/total depth of the allele at a particular locus.

To profile the mutations, we compared the mutations from tumors under different immune selection and enriched hotspot mutations as rules showed in Table 2.

**Table 2:** Filter criteria for hotspot mutation profiling.

Category/group	In vitro	Immunodeficient	Immunocompetent	Immunotherapy	
		Nude	C57BL/6 J	αPD-L1	αPD-1
Category 1:PD-1 dependent immune suppressor candidates	Negative	Negative	Positive in at least two of these tumors	Negative	
Category 2:Non-PD-1 immune suppressor candidates	Negative	Negative	Positive in at least two of these tumors	Positive in any tumor	

(Positive, AF ≥ 0.1; Negative, AF < 0.1)

### mMCE pooled sgRNA library construction

The oligonucleotide pool targeting 53 candidate genes from profiled heterogeneity and controls (ten individual sgRNAs per gene) was customized at CustomArray, Inc. Then, the oligo pool was annealed and subcloned into lentiCRISPRv2 (Addgene, 52961) backbone using the Gibson Assembly Kit (NEB) and electroporation according to the protocol from Zhang’s lab^72^. Library representation was maintained for at least 1000× coverage at each step of the process. To qualify the sgRNA representation, the constructed library was amplified by two-step PCRs using Phusion® Hot Start Flex DNA Polymerase (NEB, M0535L), in which the second step of PCR was conducted using the PCR amplicon from the first PCR as template. The primers for the first step of PCR are 5′-AATGGACTATCATATGCTTACCGTAACTTGAAAGTATTTCG-3′ (Forward) and 5′-TAGGCACCGGATCAATTGCCGAC-3′ (Reverse). The primers for the step of PCR are 5′-(1–9 bp of variable length sequence)TCTTGTGGAAAGGACGAAACACCG-3′ (Forward) and 5′-(1–9 bp of variable length sequence)TGTGGGCGATGTGCGCTCT-3′ (Reverse). The amplicon was sent for next-generation sequencing (NGS) at Mingma Technologies using illumina Hiseq X10 (3.3 M reads, 1 G data) to qualify sgRNA library distribution before screening^72^.

### Lentivirus production

To produce lentivirus, 3 × 10^7^ 293FT cells were collected for reverse-transfection in each T150 flask. For each T150 flask, 15 µg library or other sgRNA plasmid, 11.25 µg psPAX2 (Addgene, 12260), and 7.5 µg pCMV-VSV-G (Addgene, 8454) diluted in 2 ml Opti-MEM (Gibco, 11058021) were mixed with 100 µL Lipofectamine 2000 (Invitrogen, 11668019) diluted in 2 ml Opti-MEM. The mixture was added into 293FT cells and the medium was refreshed 12 h post transfection. Virus supernatant was collected 48 h post refreshing medium, centrifugated at 1, 800 g at 4 °C for 10 min to pellet the cell debris and filtrated with a 0.45 µm low protein binding membrane (Millipore, SE1M003M00). The lentivirus was concentrated by ultra-centrifugation at 25,000 rpm at 4 °C for 2 h, and then resuspended and aliquoted to store at −80 °C.

### Lentivirus infection

To test the lentivirus titer, MC38 or MC38-OVA cells were seeded at 5 × 10^5^/well in 6-well plates and infected by lentivirus at gradient dilution with polybrene (7.5 µg/mL) added 24 h later. 24 h post infection, cells were collected and re-seeded (1 × 10^3^ cells/well in 96-well plates) with 6 replicates for each dose. The next day, according to the vector backbones, puromycin (Gibco, A1113803), hygromycin B (Invitrogen, 10687010), or G418 (Gibco, 10131027) was added to 3 of 6 replicated wells at 2.5 µg/ml, 100 µg/ml or 500 µg/ml, respectively, for 7 days and then cell viability was detected by CellTiter-Glo Kit (Promega, G7570).

According to the virus titer, concentrated lentivirus infection at a low MOI (~0.3) was conducted for MC38 or MC38-OVA cells with polybrene added. The transduced cells were treated with puromycin, hygromycin B or G418 starting from 2 days post infection and kept selection for 7 days. Selected cells were maintained with a low concentration of puromycin (0.5 µg/ml), hygromycin B (50 µg/ml) or G418 (200 µg/ml). Antibiotics were removed 2 days before tests.

### In vivo CRISPR screen

mMCE sgRNA library was transduced into MC38 cells for tumorigenesis in nude or C57BL/6 J mice with different treatments to validate profiled tumor heterogeneity. Together with inoculation, 2 × 10^6^ cultured MC38 cells with mMCE library were collected as in vitro baseline. Tumors were randomly divided into the following groups, nude (*n* = 8) mice or WT mice with IgG (*n* = 8), anti-PD-1 (*n* = 10), anti-PD-L1 (*n* = 10), or anti-CD4/8 (*n* = 7), for treatment as described. Before treatment, five additional WT tumors were collected as in vivo baseline. And, 9 days after treatment, tumors (volume = 50~1500 mm^3^) were collected and grinded into homogeneous powders in liquid nitrogen. Genomic DNA was extracted by Blood & Cell Culture DNA Midi Kit (QIAGEN,13343) from the powders. PCR amplification was conducted using 1 µg of the genomic DNA as a template following the protocol for sgRNA library plasmid NGS sampling^72^. For each sample, 6 of 50 µl-reactions were performed for the first step of PCR and 2 of 50 µl-reactions were performed for the second step of PCR. Paired-end 150 bp NGS was performed on the amplicons from the second PCR using an Illumina Hiseq X10 to determine sgRNA abundance (3.3 M reads, 1 G data per sample).

### T cell co-culture screen and assay

OT-I T cells were isolated from spleen and lymph nodes of OT-I transgenic mouse and stimulated with 2 ng/mL recombinant mouse IL-2 (Novoprotein, CK24), 5 ng/mL mouse IL-7 (Peperotech, 217-17) and 100 ng/mL mouse IL-15 (Peperotech, 210-15) for 2 days. Then, T cells were co-cultured with tumor cells stimulated with 1 µM SIINFEKL peptide (OVA p257-264, GL Biochem, 53,698) for 2 h or tumor cells stably expressing OVA peptide in the complete T cell medium with 2 ng/mL mouse IL-2 added.

For T cell co-culture screen, MC38-OVA cells transduced with mMCE sgRNA library post puromycin selection were seeded at 2 × 10^6^ cells (library coverage > 2000×) in each T750 flask (total 12 flasks). The next day, 2 × 10^6^ effector T cells were planted in each flask with 2 ng/mL mouse IL-2 added. 4 × 10^6^ MC38-OVA cells (library coverage > 4000×) were collected as the baseline. With no-T cells as the negative control, tumor cells were collected at day 1 (10 × 10^6^ cells), day 2 (4 × 10^6^ cells) and day 2.5 (1–2 × 10^6^ cells, library coverage > 1000×) post co-culture for NGS as described in the in vivo screen (*n* = 3 for each group per day).

For T cell co-culture assay, 200 genome-edited OVA expressing or treated (1 µM for 2 h) tumor cells were plated onto 96-well plates and co-cultured with extracted T cells for 2 days. T cells were analyzed by flow cytometry. Tumor cells post co-culture were analyzed by CellTiter-Glo Kit (Promega, G7570) for cell viability.

### Data process and analysis for CRISPR screen

MAGeCKFlute was used to generate read counts for each sgRNA based on fastq files from paired-end NGS of PCR amplicons^73^. In order to ensure reliable ranking in depletion analysis, we tried to filter sgRNAs with low reads which could be a consequence of insufficient sampling of NGS. The mapped reads from all samples are over 30% (from 30.65% to 34.43% for in vivo screen, and from 33.88% to 34.43% for T cell co-culture screen, respectively) for PE reads, which means over 60% for one side covering the sgRNA region in the PCR product. In the filtering step for sgRNAs with low counts, we found no sgRNA with zero count. Both mapping rates and zero-count percentage demonstrate the high quality of each sample’s NGS data for both the in vivo and in vitro screen. Then, the read counts for each sgRNA were normalized according to the formula as follows: Normalized reads per sgRNA =$\frac{{{{{\rm{Reads}}}}}\; {{{{\rm{per}}}}}\; {sgRNA}} {{Total\; count\; of\; mapped\; reads}}$ × (Max mapped reads count among all samples) + 1. Tumor-intrinsic immune evasion gene hits were determined based on both integrated beta score ranking (generated by MAGeCK-RRA^73^) and log_2_ fold-change (generated by edgeR^74^) with relevant *P* value (< 0.05) in the comparisons of immunocompetent vs immunodeficient from in vivo screen and OT-I T cell *vs* No T cell from T cell co-culture screen.

### Construction of genome-engineered cell lines

MC38 and MC38-OVA cells with sgRNA targeting *Ankrd52*, *Ppp6c*, *Ago2*, *Dicer1*, *Xpo5,* or non-targeting control were generated using lentiCRISPRv2 (Addgene, 52961) vectors harboring annealed relevant sgRNA oligonucleotides. The sgRNA vectors were packaged into lentivirus and infected the mentioned cell lines (MOI ≈ 0.3). Cells with sgRNAs were selected with 2.5 µg/ml puromycin (Gibco, A1113803) for 72 h and maintained with a low concentration of puromycin (0.5 µg/ml). Knockout efficiency was evaluated by WB analysis. Base on it, *Ankrd52*-null or control MC38 cells were generated from MC38 cells with sgRNA targeting *Ankrd52* or non-targeting control by limiting dilution and single-cell clonal expansion. Confirmation of gene knockout was performed using QIAamp DNA Blood Mini kit (Qiagen), and sgRNA target regions were amplified by PCR followed by Sanger sequencing for frame-shift mutations. CRISPR-mediated *Ankrd52* knockouts were also validated by RNA-Seq reads at the sgRNA targeting sites. Meanwhile, *ANKRD52*-null 293 T cells were generated using px459 (Addgene, 62988) vectors introduced with sgRNA targeting ANKRD52. 293 T cells were transfected with the sgRNA vector by Lipofectamine 3000 reagent (Invitrogen) and selected with puromycin (1 µg/ml) for 72 h. Selected cells were subjected to limiting dilution for single-cell clonal expansion and confirmation of knockout. Moreover, *Ankrd52*-null or control MC38 cells with sgRNA targeting *Socs1* or non-targeting control were generated using pLVX-hyg-sgRNA (TaKaRa, 632632) vectors with relevant sgRNA by hygromycin B (Invitrogen, 10687010) selection following viral infection (MOI ≈ 0.3). All the sgRNA sequences are listed in Supplementary Data 4.

*Ankrd52* mutant or recombinant cells were generated by stably overexpressing WT or clinic hotspot mutant *Ankrd52* in *Ankrd52*-null MC38 cells. The WT cDNA sequences for *Ankrd52* were designed with sgRNA-targeting-defective synonymous mutations and a FLAG-tag added at N-terminal, and synthesized at GENEWIZ. The cDNA was subcloned into pLVX-IRES-Neo vector (Clontech, 632181) and introduced with clinic hotspot mutations using Phusion Site-Directed Mutagenesis Kit (Thermo Fish, F541). *Ankrd52*-null MC38 cells were introduced with the cDNA vectors, respectively, by lentivirus (MOI ≈ 0.3) and selected by G418 (Gibco, 10131027).

### In vivo competition assay

MC38 cells were transduced with four individual sgRNAs targeting *Ankrd52* and three individual sgRNAs targeting non-targeting control. These sgRNA cells were mixed in equal proportions and inoculated into nude or C57BL/6 J mice. Mice were euthanized 16 days after tumor inoculation for tumor collection when the mean tumor volume in nude mice reached 1000 mm^3^. Tumor were grinded into homogeneous powders in liquid nitrogen for genomic DNA extraction and NGS. sgRNA distribution was analyzed by edgeR for log_2_ fold-change^74^.

### Analysis of tumor-infiltrating immune cell populations

*Ankrd52*-null and control tumors were harvested at ~1000 mm^3^ and cut up on ice for the following incubation in collagenase D (1 mg/ml, Roche), DNase I (50 µg /ml, Sigma-Aldrich) and Hyaluronidase (100 µg/mL, Sigma-Aldrich) supplemented RPMI-1640 (Gibco) for 30 min at 37 °C. After incubation, tumor cells were passed through 70-µm filters to remove the undigested tumor. Then, these single tumor cell suspensions were washed with ice-cold PBS with 2% FBS and stained with Live/Dead (1:1000, Invitrogen) combined with antibodies for 30 min at 4 °C. BD LSRFortessa X-20 was used for data acquisition and FlowJo (10.4) was used for data analysis.

### Western blot

Cell pellets were lysed by RIPA buffer (Sigma, R0278) with a complete EDTA-free protease inhibitor cocktail (Roche, 11836170001). Protein concentrations were quantified with the Pierce BCA Protein Assay Kit (Thermo Fisher). Equal amounts of protein (10–20 µg) were run with SDS–PAGE for separation on 4–12% Bis-Tris NuPage gels or 3–8% Tris-Acetate NuPage gels (Thermo Fisher). Protein was transferred to 0.45 µm PVDF membranes (Merck). Membranes were blocked in Tris-buffered saline plus 0.1% Tween 20 (TBST) dissolved 5% non-fat dry milk for 1 h at room temperature followed by overnight incubation with primary antibodies at 4 °C. Membranes were washed by TBST and incubated with HRP-conjugated secondary antibodies for 1 h at room temperature. HRP was activated with Supersignal West Dura Extended Duration Substrate (Pierce) and visualized with ChemiDoc Imaging System (Bio-Rad).

### Phos-tag SDS–PAGE electrophoresis

To measure phosphorylation of AGO2, SDS–PAGE gels (7%) were supplemented with Phos-tag AAL solution (Wako) according to the manufacturer’s recommendations. Gels after SDS–PAGE running were incubated in transfer buffer supplemented with 1 mM EDTA for 20 min, and then soaked in normal transfer buffer for 10 min. Proteins were transferred to a nitrocellulose membrane and standard western blotting procedures were subsequently followed.

### Antibodies and compounds

For western blot, primary antibodies against mouse ANKRD52 (Santa Cruz, sc-398544) and CK1*a* (Santa Cruz, sc-6477) were used with dilution as 1:100; human ANKRD52 (Bethyl, A302-372A), OVA (Sigma, SAB5300165), β-Actin (CST, 4967), JAK1 (CST, 3332), p-STAT1 (Tyr701) (CST, 9167), p-STAT3 (Tyr705) (CST, 9145), SOCS3 (CST, 52113), TAP1 (CST, 12341), FLAG (Sigma, F3165), Vinculin (Sigma, v9131), PPP6C (Abcam, EPR8764), AGO2 (CST, 2897 S), XPO5 (CST, 12565 S), and DGCR8 (Abcam, ab191875; Proteintech, 10996-1-AP) were used with dilution as 1:1000. Londaysin (S657801) purchased from Selleck Chem were dissolved in DMSO (Thermo Fisher).

For flow cytometry, the following anti-mouse fluorochrome-conjugated antibodies were used with dilution as 1:100: AF700 anti-CD45 (560510), PerCP-Cy5.5 anti-CD4 (550954), BV510 anti-CD8 (563068) and BV605 anti-CD44 (563058), BV605 anti-CD25 (563061) purchased from BD Biosciences; PE anti-H2K^b^/H2D^b^ (114607), APC anti-H2K^b^ bound to SIINFEKL (141605), FITC anti-Lag-3 (369308), FITC anti-CD69 (104506) and AF594 anti-GZMB (372216) purchased from BioLegend; PerCP-Cy5.5 anti-CD274 (46-5982-82), PE-Cy7 anti-FoxP3 (25-5773-82) and PE anti-Tim-3 (12-5870-82) purchased from ebioscience.

### Antigen presentation analysis

OVA-treated (1 µM for 2 h) MC38 cells were simulated with 10 ng/ml IFNγ (CST, 39127) for 24 h. Then tumor cells were trypsinized and washed in PBS + 2% FBS, stained with antibodies against cell surface H2K^b^ and H2K^b^ bound to SIINFEKL as the manufacturer’s instructions and then analyzed on an BD LSRFortessa X-20 flow cytometry system. FlowJo (10.4) was used for data analysis.

### Quantitative RT-PCR

Total RNA was isolated with TRIzol Reagent (invitrogen, 15596026) from tumor cells as Invitrogen user guide. cDNAs were synthesized from 1 µg of total RNA using the SPARKscritpt II RT Plus Kit (SparkJade) and were amplified by 2 × SYBR Green qPCR Mix (SparkJade) using Quantstudio 7 Real-Time PCR System (Life Technologies) according to the manufacturer’s protocols. Relative mRNA expression was evaluated after normalization for Gapdh expression. Each experiment was performed in triplicate. The primers used for quantitative RT-PCR are listed in Supplementary Data 5.

### Luciferase reporter assay

psiCheck2 luciferase reporter plasmids (Promega) containing WT or mutated SOCS1 3′ UTR and miR155-expressing or empty pMDH-PGK-EGFP plasmids were gifts from Dr. Li-Fan Lu at UCSD^48^. These plasmids were co-transfected into 293 T cells (which were seeded at 5 × 10^5^ cells/well in 6-well plates 1 day prior to transfection) using Lipofectamine 3000 reagent (Invitrogen). Cells were harvested 24 h later, and luciferase activity was assessed with the Dual-Luciferase Reporter Assay System (Promega) according to the manufacturer’s protocol.

### TCGA dataset analysis

The somatic mutation data inferred by MuTect2, mRNA expression (FPKM) and clinical survival data for 33 TCGA cohorts were downloaded from UCSC Xena (GDC TCGA, https://gdc.xenahubs.net) with R package UCSCXenaTools^75^. The Spearman correlation between *ANKRD52* or miRNA machinery expression and *SOCS1* in TCGA dataset was calculated by TIMER^49^. The Spearman correlation between *ANKRD52* or miRNA machinery expression and T cell infiltration in TCGA dataset was calculated by EPIC^76^, which were reported as more suitable for CD4^+^ and CD8^+^ T cell infiltration analysis^77^. The impact of mutations in miRNA machinery on survival was shown for patients whose tumors had higher (> 75%) or lower (< 75%) infiltration score of cytotoxic CD8 T cells (mean of CD8A, CD8B, GZMB, and PRF1)^9^. All of TCGA analysis data (containing the results of TIMER and EPIC analysis) were obtained from TIMER2.0 database^49^ (http://timer.cistrome.org).

### Statistical analysis

For all experiments, the number of technical and/or biological replicates is provided in the figure legends or text. Microsoft Excel (16.27) was used to organize data into tables. In all cases, ****P < 0.0001, ***P < 0.001, **P < 0.01, *P < 0.05. Statistical analyses were performed using Graph-Pad Prism (8.2.1) or the R language (3.6.0) programming environment using RStudio (1.2.1335) to calculate the ‘*p*-value’’. In CRISPR screen or RNA-Seq analysis, the expression matrix is modeled using an overdispersed poisson model in EdgeR after above-mentioned normalization. Gene dispersions are then estimated by conditional maximum likelihood and shrunk using an empirical Bayes procedure. Finally, differential expression is assessed using an adapted Fisher’s exact test and get the ‘*p*-value’’^74^. In MAGeCK RRA software, just like in EdgeR, the ‘*p*-value’’ is calculated by randomly permuting sgRNA labels. A negative binomial (NB) model is used to test whether sgRNA abundance differs significantly between treatments and controls and a modified robust ranking aggregation (RRA) algorithm is used to identify positively or negatively selected genes based on *p*-value calculated from the NB model^73^. In GSEA, the ‘*p*-value’’ is calculated by the permutation test. All ‘Spearman’s correlation’ with ‘*p*-value’’ data were obtained from Depmap or TIMER 2.0 database as mentioned above.

### Reporting Summary

Further information on research design is available in the Nature Research Reporting Summary linked to this article.

## Supplementary information

Supplementary Information

Supplementary Dataset 1

Supplementary Dataset 2

Supplementary Dataset 3

Supplementary Dataset 4

Supplementary Dataset 5

Description of Additional Supplementary Files

Reporting Summary

## Data Availability

The raw FASTQ files and the source datasets generated and analyzed in this study for the sequencing data are available in the Genome Sequence Archive (GSA) database with accession number CRA004140, CRA004141, CRA004145, CRA004146). Descriptions of the analyses, tools and algorithms are provided in the Methods or GSA. The TCGA mutation data and analysis data used in this study are available in the UCSC Xena database and TIMER2.0 database, respectively. The remaining data are available within the Article, Supplementary Information or Source Data file. Source data are provided with this paper.

## Source data

Source Data
