# Supplementary material for: Tumor evolution selectively inactivates the core microRNA machinery for immune evasion
Source: Nat Commun. 2021 Dec 1;12:7003. doi: 10.1038/s41467-021-27331-3 (PMC8636623; doi:10.1038/s41467-021-27331-3)
Supplement: Supplementary file 1 — Supplementary Information [file 41467_2021_27331_MOESM1_ESM.pdf]

Supplementary Figures

Supplementary Fig. 1

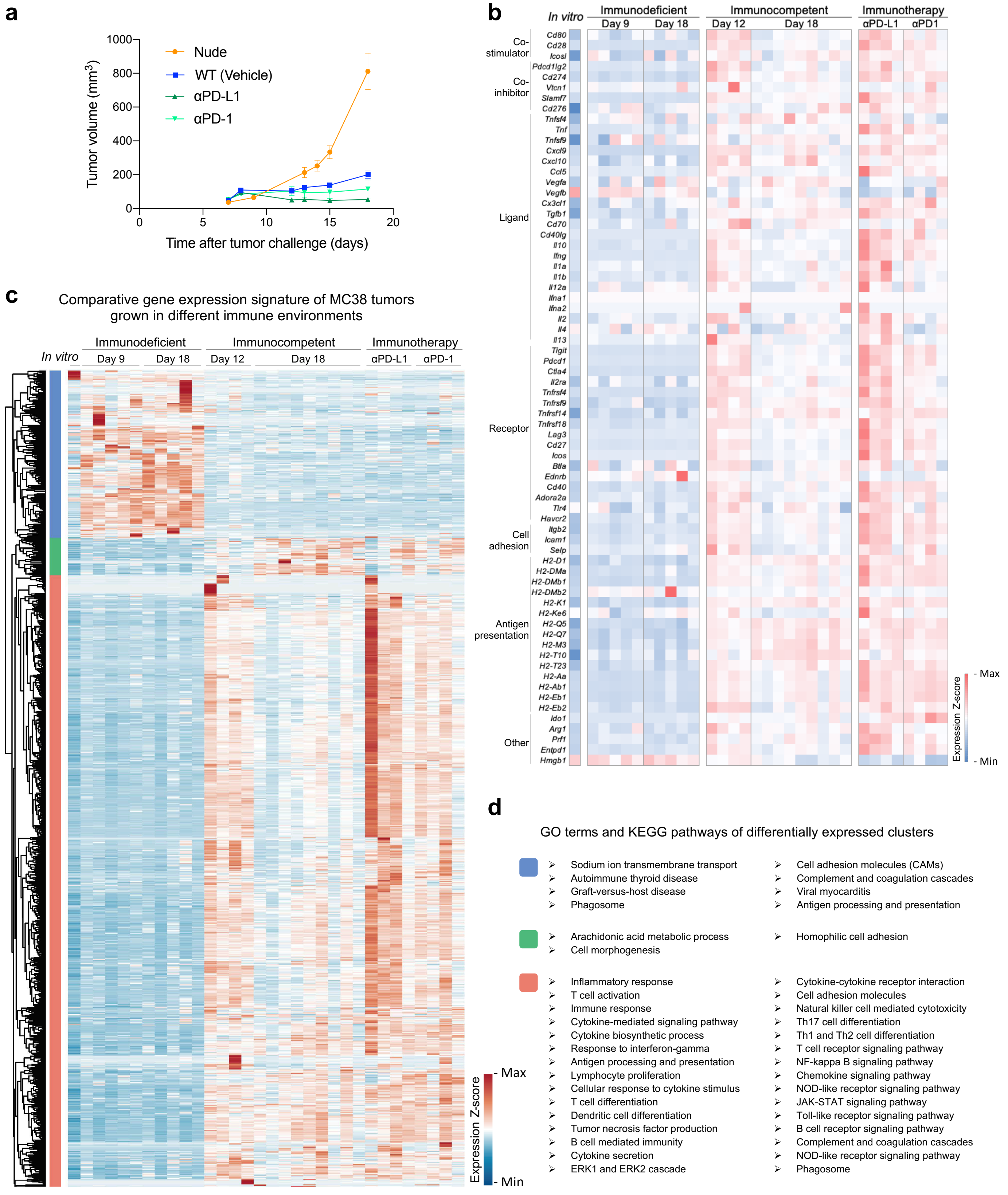

Supplementary Fig. 1 | Profiling of tumor heterogeneity under different levels of immune selection. Related to Fig. 1. Source data are provided as a Source Data file.

a, *In vivo* tumor growth curve of MC38 cell line-derived tumors grown under different levels of T cell pressure (n = 10 for nude mice; n = 13 for WT mice; n = 8 for WT mice with αPD-1/αPD-L1 treatment). Mean ± s.e.m.

b, Expression signature of stimulatory and inhibitory immune modulators in MC38 tumors.

c, Unsupervised cluster analysis of differentially expressed genes in MC38 tumors (2-fold-change cutoff, $P < 0.05$).

d, Gene ontology and KEGG pathway analysis of differentially expressed gene clusters (colors correspond to clusters in (**c**), $P < 0.01$ for cluster 1 and 2, $P < 0.001$ for cluster 3).

Supplementary Fig. 2

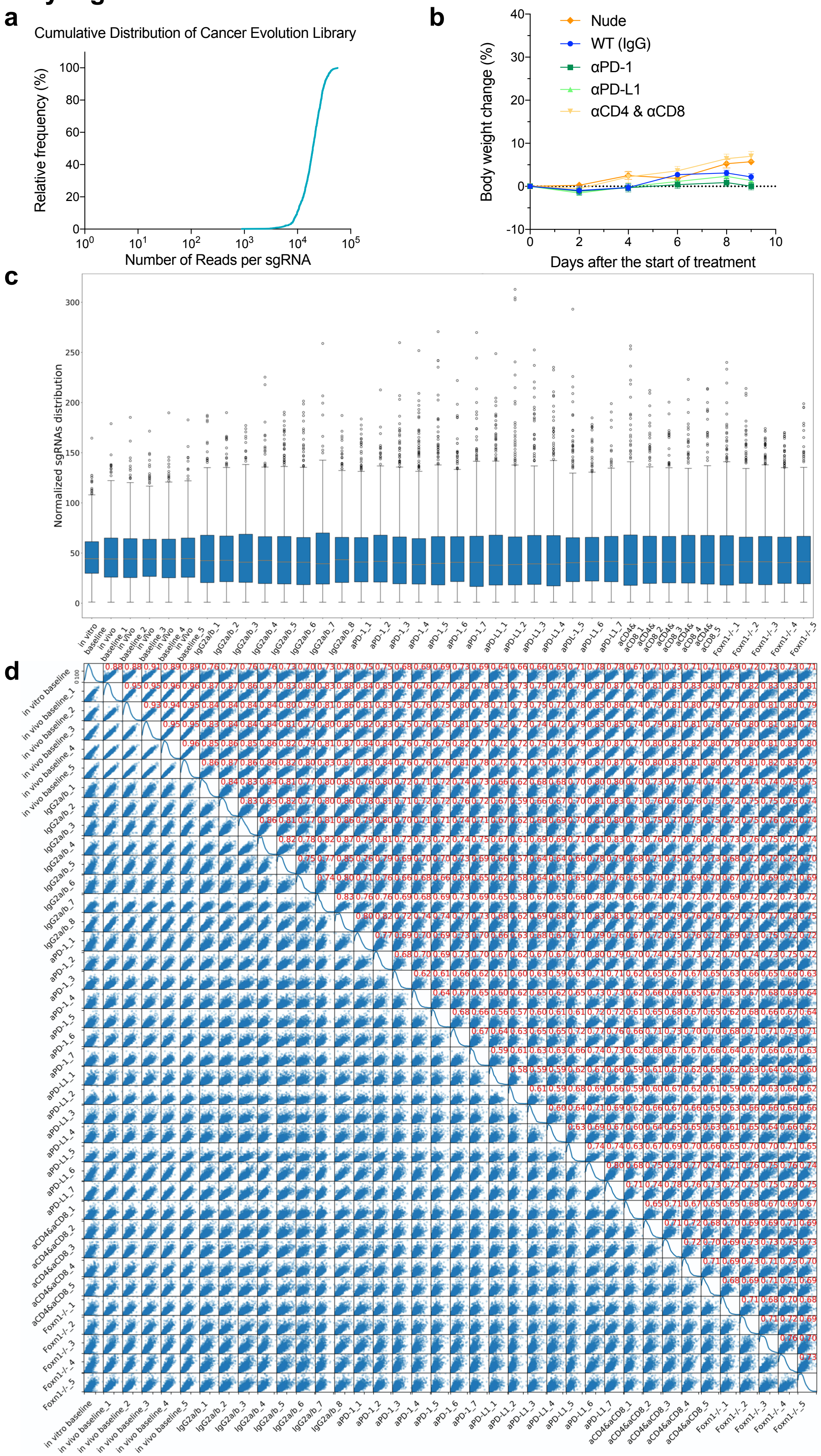

Supplementary Fig. 2 | Quality control of *in vivo* CRISPR screen to validate candidates from profiled tumor heterogeneity. Related to Fig. 2. Source data are provided as a Source Data file.

a, Cumulative distribution of the read counts per sgRNA in the mMCE library targeting candidates from profiled tumor heterogeneity.

b, *In vivo* body weight change curve in the *in vivo* screen for groups indicated (n = 8 for WT mice with rat IgG2a and IgG2b isotype treatment (WT); n = 10 for WT mice with PD-1 or PD-L1 antibodies treatment (αPD-1 or αPD-L1); n = 8 for nude mice with rat IgG2a and IgG2b isotype treatment (nude) and n = 7 for WT mice with CD4 and CD8 treatment (αCD4 & αCD8)). Mean ± s.e.m. Not significant for comparison of every two group (two-way analysis of variance (ANOVA)).

c, Boxplot showing distribution of the normalized reads for sgRNA library pool of each sample in the *in vivo* screen analyzed by MAGeCK. IgG2a/b represents WT tumor, *Foxn1*$^{-/-}$ represents nude tumor (n = 1 for *in vitro* baseline, n = 5 for *in vivo* baseline tumor, n = 8 for WT tumor; n = 7 for tumor with αPD-1 or αPD-L1 treated tumor; n = 5 for nude tumor and n = 5 for αCD4 & αCD8 tumor).

d, A matrix with Pearson's correlation coefficient showing the comparison of sgRNA library distribution among different tumor samples. The squares of lower left half show the correlation of comparison in pairs while the right upper half shows the coefficient in number ($P < 0.05$, analyzed by MAGeCK).

Supplementary Fig. 3

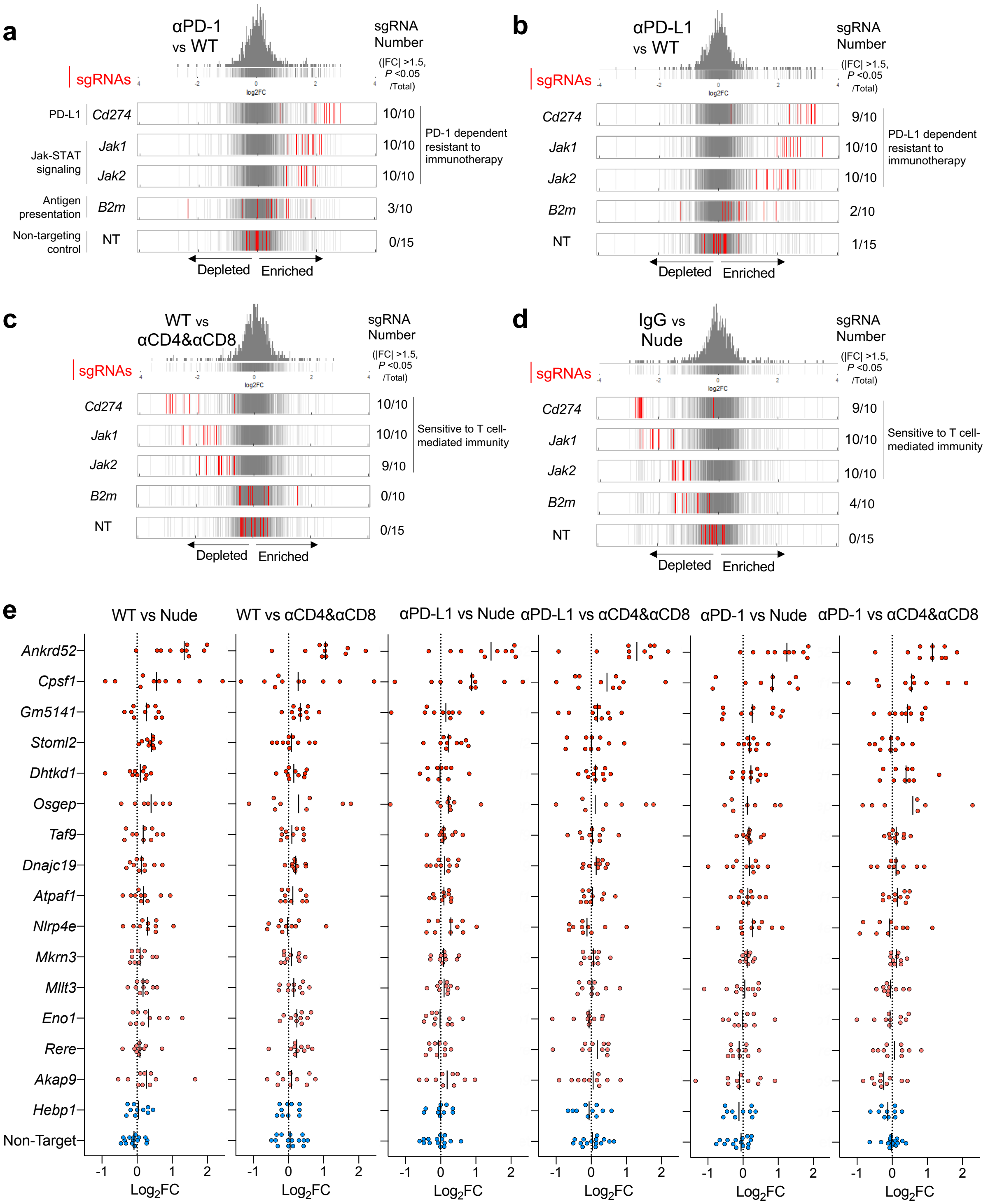

Supplementary Fig. 3 | *In vivo* CRISPR screen identifies ANKRD52 as a novel cancer immunity regulator. Related to Fig. 2. Source data are provided as a Source Data file.

a–**d**, Distribution of $\log_2$FC values for sgRNAs in the comparisons of anti-PD-1 vs WT (**a**), anti-PD-L1 vs WT (**b**), WT vs anti-CD4 and anti-CD8 (**c**), and WT vs nude (**d**). Log_2FC for 10 individual sgRNAs targeting

Cd274 (PD-L1), *Jak1*, *Jak2*, *B2m* and non-target control are demarcated in red lines, overlaid on gray gradient depicting the overall distribution. Enriched or depleted sgRNA number for marked genes in the comparisons is shown on the right side (Cutoff: |FC| >1.5, $P < 0.05$, edgeR).

e, Distribution of $\log_2$ Fold-change (FC) for sgRNAs targeting representative candidate genes from tumor profiling.

Supplementary Fig. 4

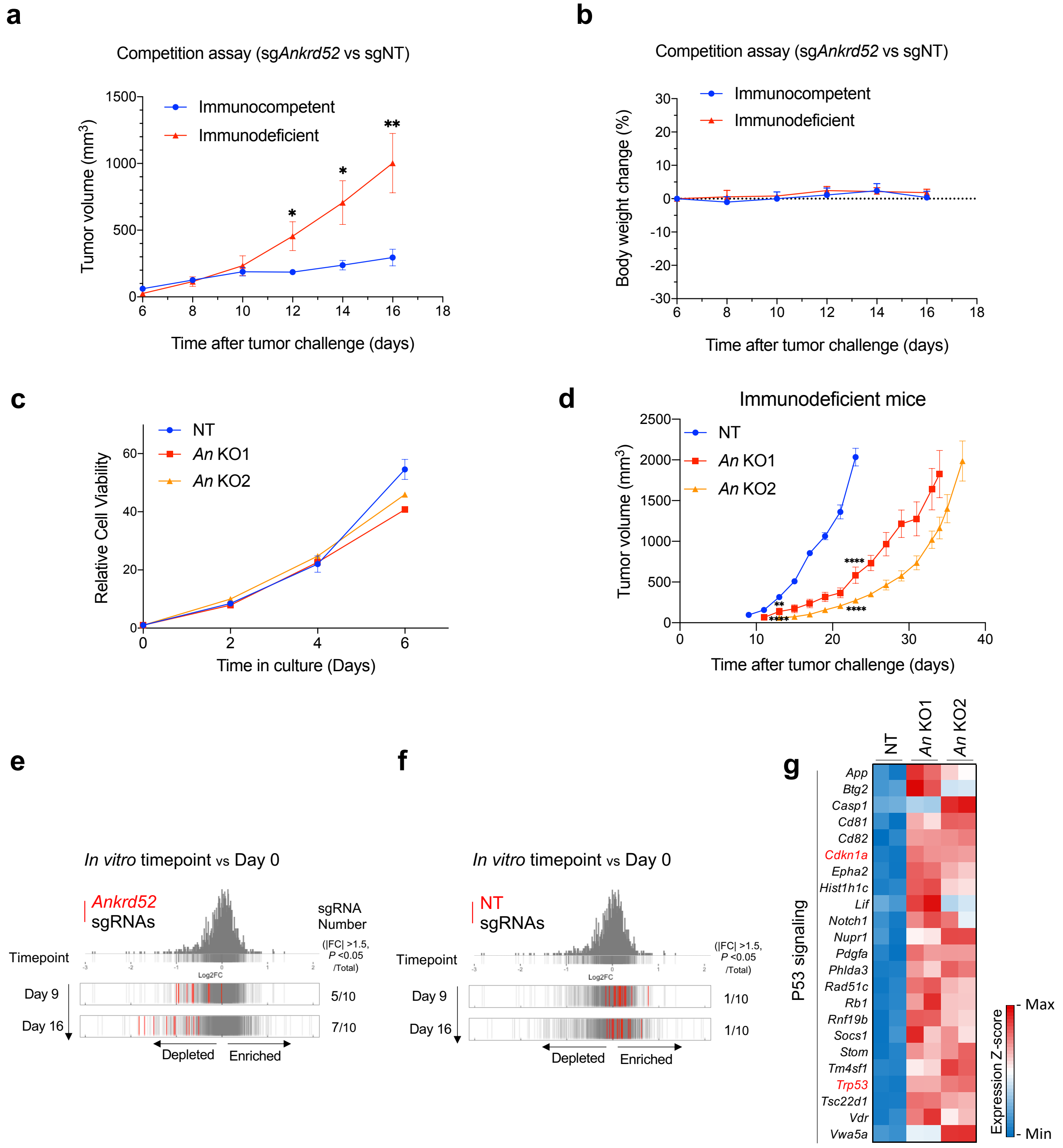

Supplementary Fig. 4 | Targeted CRISPR screen identifies ANKRD52 endowing tumor *in-vivo* growth disadvantages under compromised T cell pressure. Related to Fig. 2. Source data are provided as a Source Data file.

a, **b**, *In vivo* tumor growth curve (**a**) and body weight change curve (**b**) of the competition assay of sgRNAs against *Ankrd52* and non-targeting control in MC38 tumors (n = 5 for immunodeficient nude mice or immunocompetent WT mice). Tumor cells with each sgRNA were mixed in equal proportions before

inoculation. *P < 0.05, **P < 0.01, multiple two-tailed Student's *t*-test (tumor volume of immunocompetent vs immunodeficient, P =0.031 for day 12, P =0.016 for day 14, P =0.009996 for day 16).

c, Cell viability of control or *Ankrd52*-null MC38 tumor cells in a 6-day culture. *An* KO1 and *An* KO2 represent two different *Ankrd52* knockout clones generated from different sgRNAs. Mean ± s.e.m., $P < 0.05$ for *An* KO1 vs NT ($n = 3$ for each group, multiple two-tailed Student's *t*-test).

d, *In vivo* tumor growth curve of *Ankrd52*-null or control MC38 tumors grown in nude mice ($n = 7$ for NT or *An* KO2, $n = 5$ for *An* KO1). Mean ± s.e.m., $P = 0.002752$ for *An* KO1 vs NT and $P < 0.000001$ for *An* KO2 vs NT on Day 13, $P = 0.000003$ for *An* KO1 vs NT and $P < 0.000001$ for *An* KO2 vs NT on Day 23 (multiple two-tailed Student's *t*-test).

e, **f**, Distribution of $\log_2$FC values for sgRNAs in a 16-day culture CRISPR screen using the Cancer Evolution library. Log_2FC for 10 individual sgRNAs targeting *Ankrd52* (**f**) and non-targeting control (**g**) are demarcated in red lines, overlaid on gray gradient depicting the overall distribution. Enriched or depleted sgRNA number for marked genes in the comparisons is shown on the right side (Cutoff: |FC| >1.5, $P < 0.05$, analyzed by edgeR).

g, Heatmap showing commonly up-regulated gene expression of p53 signaling in RNA-seq analysis of *Ankrd52*-null MC38 cells ($n = 2$ for each group, cutoff: $P < 0.05$, analyzed by edgeR).

Supplementary Fig. 5

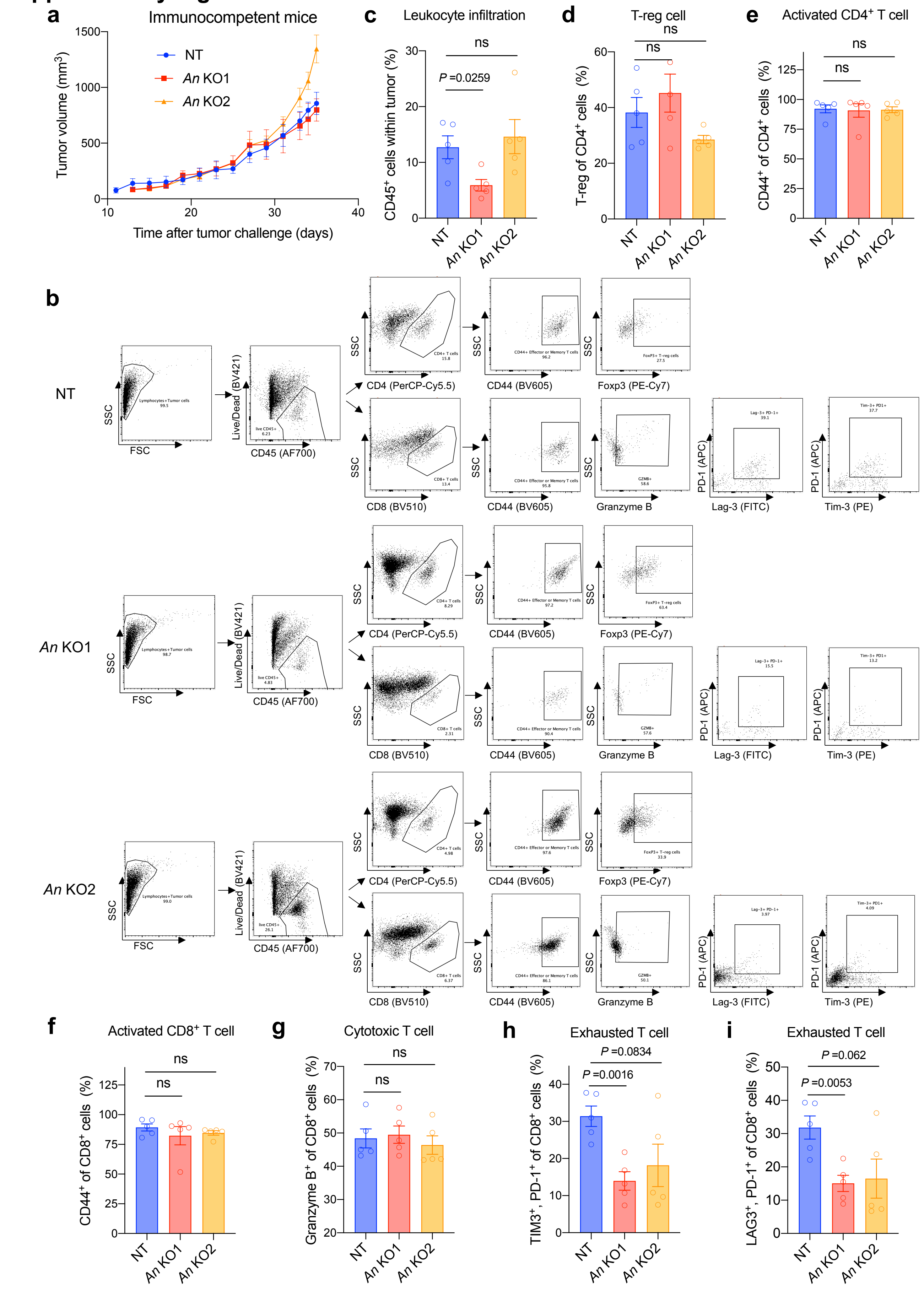

Supplementary Fig. 5 | Tumor microenvironment in *Ankrd52*-null tumors. Related to Fig. 2. Source data are provided as a Source Data file.

a, *In vivo* tumor growth curve of *Ankrd52*-null or control MC38 tumor cells in immunocompetent WT mice (n = 5 per group). Mean ± s.e.m.; not significant for *An* KO1 vs NT and *An* KO2 vs NT (two-way ANOVA).

b, Representative plots showing the gating strategy for the analysis on T cell populations in *Ankrd52*-null tumors.

c–**i**, Flow cytometry analysis of cell populations of $CD45^+$ leukocytes (**c**), $Foxp3^+$ $CD4^+$ T-reg cells (**d**), $CD44^+$ effector or memory $CD4^+$ T cells (**e**), $CD44^+$ effector or memory $CD8^+$ T cells (**f**), cytotoxic $CD8^+$ T cells (**g**), $LAG3^+$ PD-1^+ exhausted T cells (**h**) and $TIM3^+$ PD-1^+ exhausted T cells (**i**) from control and *Ankrd52*-null tumors (n = 5 per group per experiment). Mean ± s.e.m.; ns, not significant (two-tailed unpaired Student's t-test).

Supplementary Fig. 6

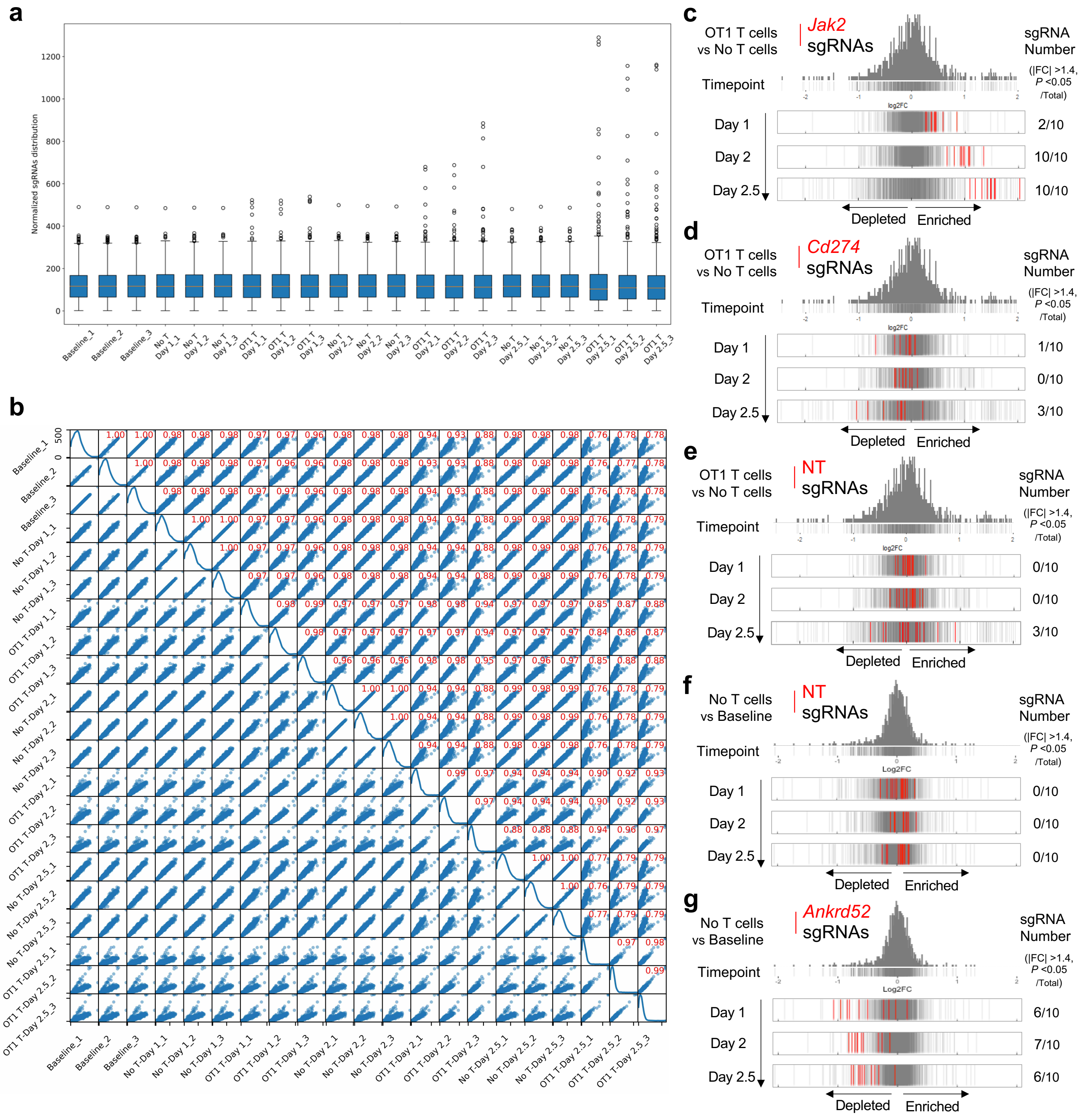

Supplementary Fig. 6 | T cell co-culture CRISPR screen identifies cancer-intrinsic regulators of T cell-mediated killing. Related to Fig. 3. Source data are provided as a Source Data file.

a, Boxplot showing distribution of the normalized reads for sgRNA library pool of each sample in the T cell co-culture CRISPR screen (n = 3 biological replicates for each group (Baseline, No T cell_Day 1, No T cell_Day 2, No T cell_Day 2.5, OT1 T cell_Day 1, OT1 T cell_Day 2 or OT1 T cell_Day 2.5)).

b, A matrix with Pearson's correlation coefficient showing the comparison of sgRNA library distribution among different MC38 cell samples of the T cell co-culture CRISPR screen. The squares of lower left half show the correlation of comparison in pairs while the right upper half shows the coefficient in number.

c–**e**, Distribution of log_2FC values for sgRNAs in the comparisons of OT-I T cells versus no T cells. Log_2FC for 10 individual sgRNAs targeting *Jak2* (**c**), *Cd274* (**d**) and non-targeting control (**e**) are demarcated in red lines, overlaid on gray gradient depicting the overall distribution. Depleted sgRNA number for marked genes at each timepoint is shown on the right side ($P < 0.05$, analyzed by edgeR).

f, **g**, Distribution of log_2FC values for sgRNAs in the comparisons of no T cells versus baseline. Log_2FC values for 10 individual sgRNAs targeting non-targeting control (**f**) and *Ankrd52* (**g**) are demarcated in red lines, overlaid on gray gradient depicting the overall distribution. Depleted sgRNA number for marked genes at each timepoint is shown on the right side ($P < 0.05$, analyzed by edgeR).

Supplementary Fig. 7

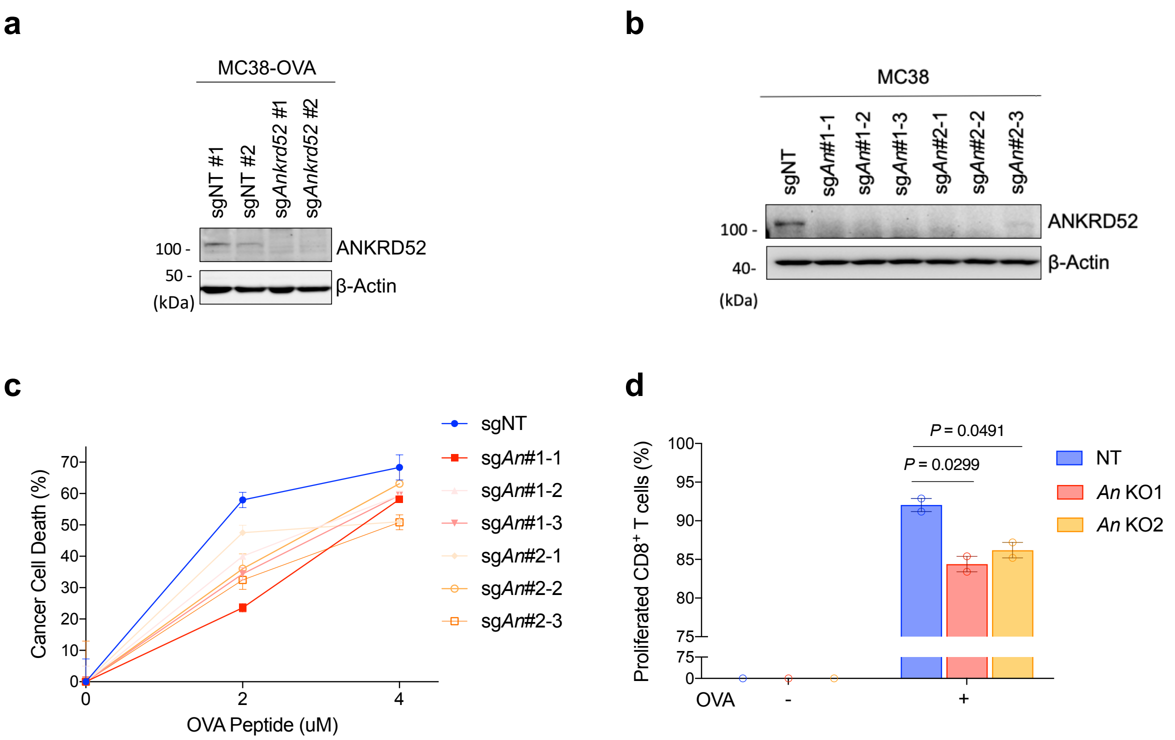

Supplementary Fig. 7 | ANKRD52 inactivation induces resistance of cancer cells to T cell-mediated cytotoxicity. Related to Fig. 3. Source data are provided as a Source Data file.

a, Protein abundance of ANKRD52 in MC38 cells stably expressing OVA antigen and transduced with sgRNA targeting NT or *Ankrd52*. Data are representative of two independent experiments.

b, Protein abundance of ANKRD52 in MC38 clones transduced with sgRNAs targeting NT or *Ankrd52*. Data are representative of two independent experiments

c, OT-I transgenic T cell-dependent killing of MC38 clones with sgRNA targeting NT or *Ankrd52* after treatment with increasing dose of OVA peptide for 2 hours (n = 3 replicates per group per condition). Mean ± s.e.m., $P < 0.05$ for 2 μM OVA treatment (sgNT vs sg*An*#1-1, 2 μM, $P = 2.42\times10^{-4}$; sgNT vs sg*An*#1-2, 2 μM, $P = 2.23\times10^{-3}$; sgNT vs sg*An*#1-3, 2 μM, $P = 1.61\times10^{-3}$; sgNT vs sg*An*#2-1, 2 μM, $P = 3.66\times10^{-2}$, 4 μM, $P = 1.36\times10^{-2}$; sgNT vs sg*An*#2-2, 2 μM, $P = 1.4\times10^{-2}$; sgNT vs sg*An*#2-3, 2 μM, $P = 2.71\times10^{-3}$, 4 μM, $P = 2\times10^{-2}$; multiple two-tailed Student's *t*-test).

d, Proliferation of $CD8^+$ T cells co-cultured with control or *Ankrd52*-null MC38 cells pre-treated with OVA peptide (1μM) for 2 hours (n = 2). Primary $CD8^+$ T cells were labeled with carboxyfluorescein succinimidyl

ester (CFSE), a dye that is only diluted with subsequent cell divisions. These T cells were then co-cultured with OVA-treated MC38 cells for 3 days to measure the proliferation calculated by the dilution of CFSE. Mean ± s.e.m., two-tailed unpaired Student's *t*-test.

Supplementary Fig. 8

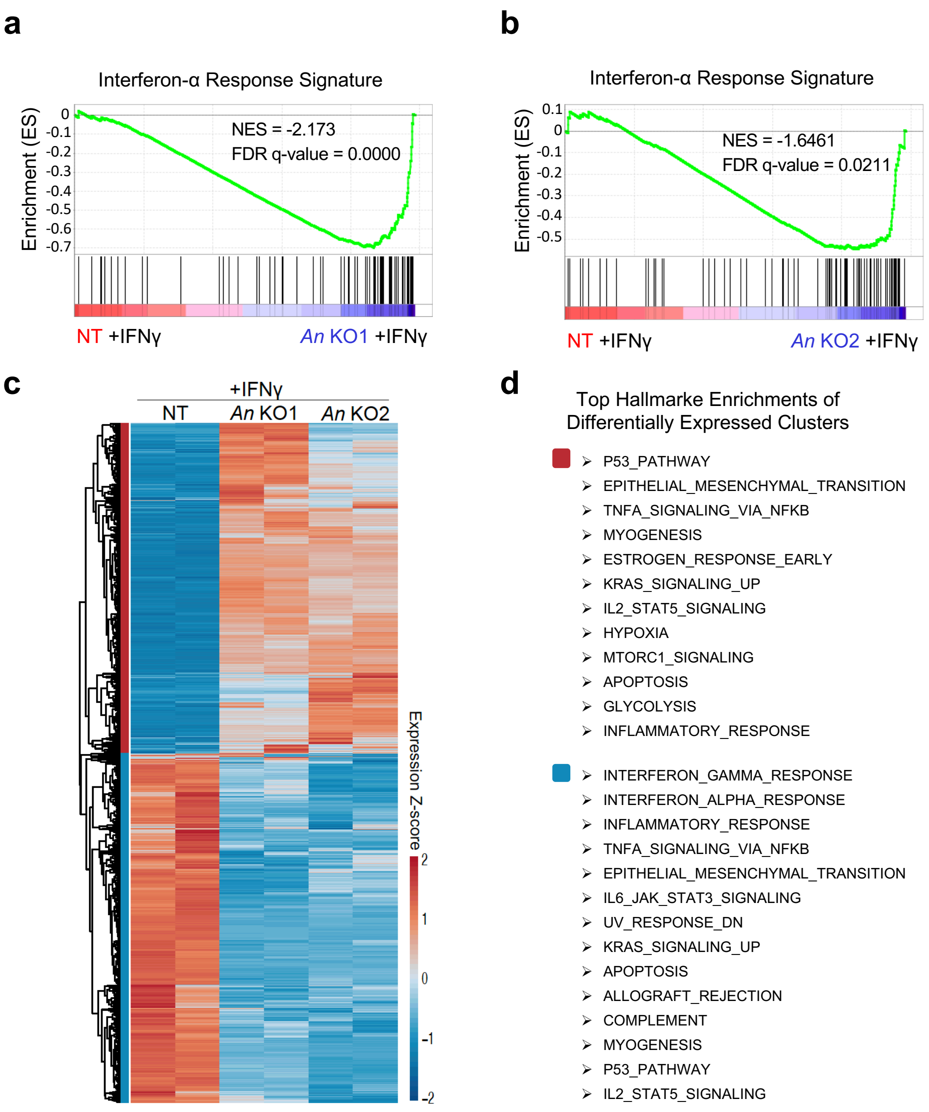

Supplementary Fig. 8 | ANKRD52 inactivation down-regulates IFN signaling. Related to Fig. 4. Source data are provided as a Source Data file.

a, **b**, Enrichment of genes associated with IFNα response in control and *Ankrd52*-null MC38 cells treated with IFNγ (10 ng/ml) for 24 hours.

c, Unsupervised cluster analysis of differentially expressed genes in *Ankrd52*-null MC38 cells treated with IFNγ (2-fold-change cutoff, FDR < 0.05).

d, Hallmark signaling analysis of differentially expressed gene clusters (colors correspond to clusters in (**c**), $P < 0.001$).

Supplementary Fig. 9

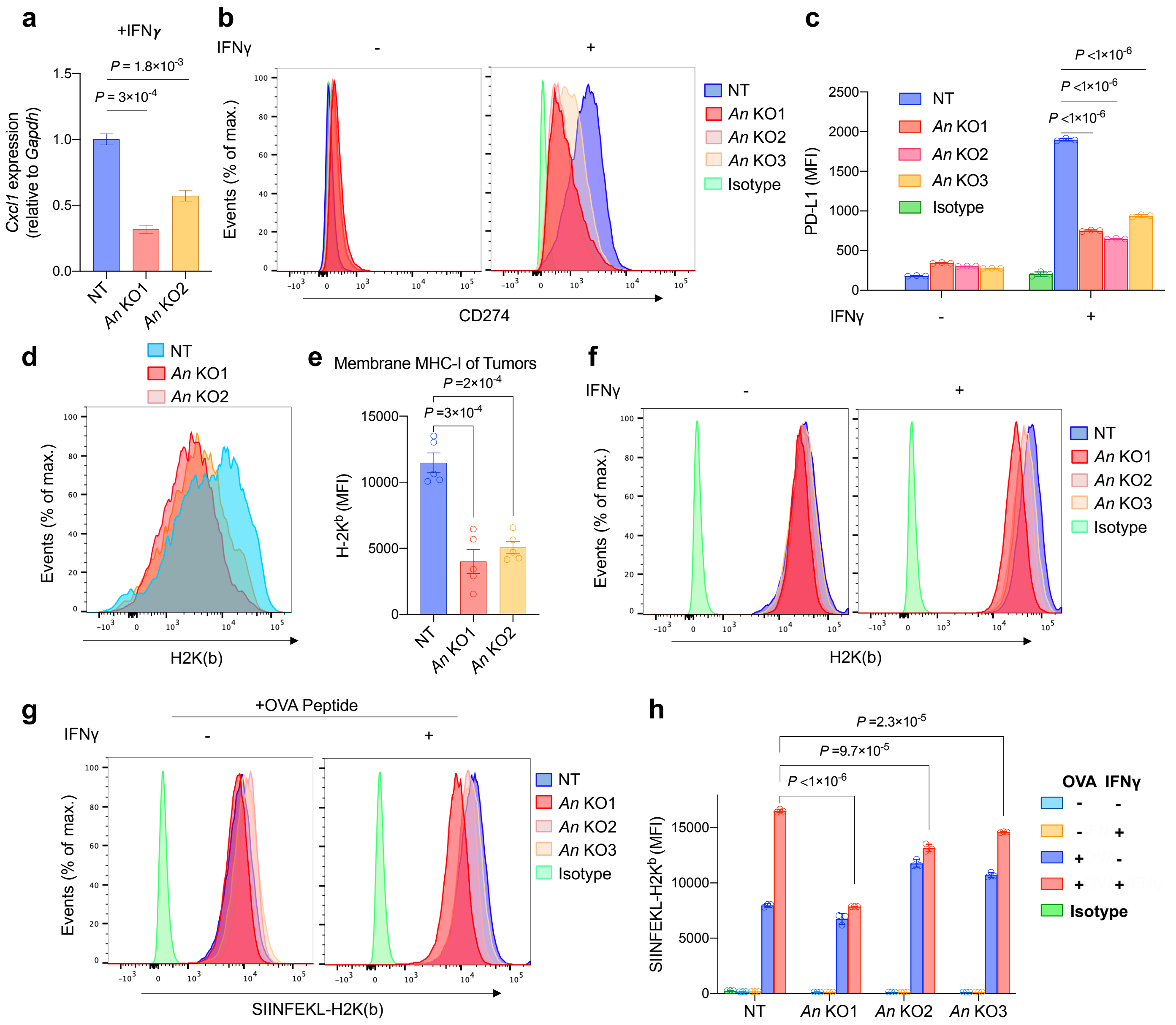

Supplementary Fig. 9 | ANKRD52 inactivation down-regulates antigen presentation. Related to Fig. 4. Source data are provided as a Source Data file.

a, *Cxcl1* mRNA level in control and *ANKRD52*-null MC38 cells treated with IFNγ (10 ng/ml) for 24 hours (n = 3 replicates per group). Mean ± s.e.m., two-tailed unpaired Student's t-test.

b, **c**, Membrane PD-L1 expression in control and *Ankrd52*-null MC38 tumor cells after treatment with IFNγ (0.5 ng/ml) or vehicle for 24 hours (n = 3 replicates per group per condition). Mean ± s.d., two-tailed unpaired Student's t-test.

d, e, Membrane MHC-I expression in control and *Ankrd52*-null MC38 tumors grown in WT mice (*n* = 5 per group). MFI, mean fluorescence intensity. Mean ± s.e.m., two-tailed unpaired Student's *t*-test.

f, Membrane MHC-I expression in control and *Ankrd52*-null MC38 tumor cells after treatment with IFNγ (0.5 ng/ml) or vehicle for 24 hours (*n* = 3 replicates per group per condition). Mean ± s.d., two-tailed unpaired Student's *t*-test.

g, **h**, Presentation of OVA-derived peptide (SIINFEKL) in control and *Ankrd52*-null MC38 tumor cells treated with OVA peptide (1μM) or vehicle for 2 hours after stimulation with IFNγ (0.5 ng/ml) or vehicle for 24 hours (*n* = 3 replicates per group per condition). Mean ± s.d., two-tailed unpaired Student's *t*-test.

Supplementary Fig. 10

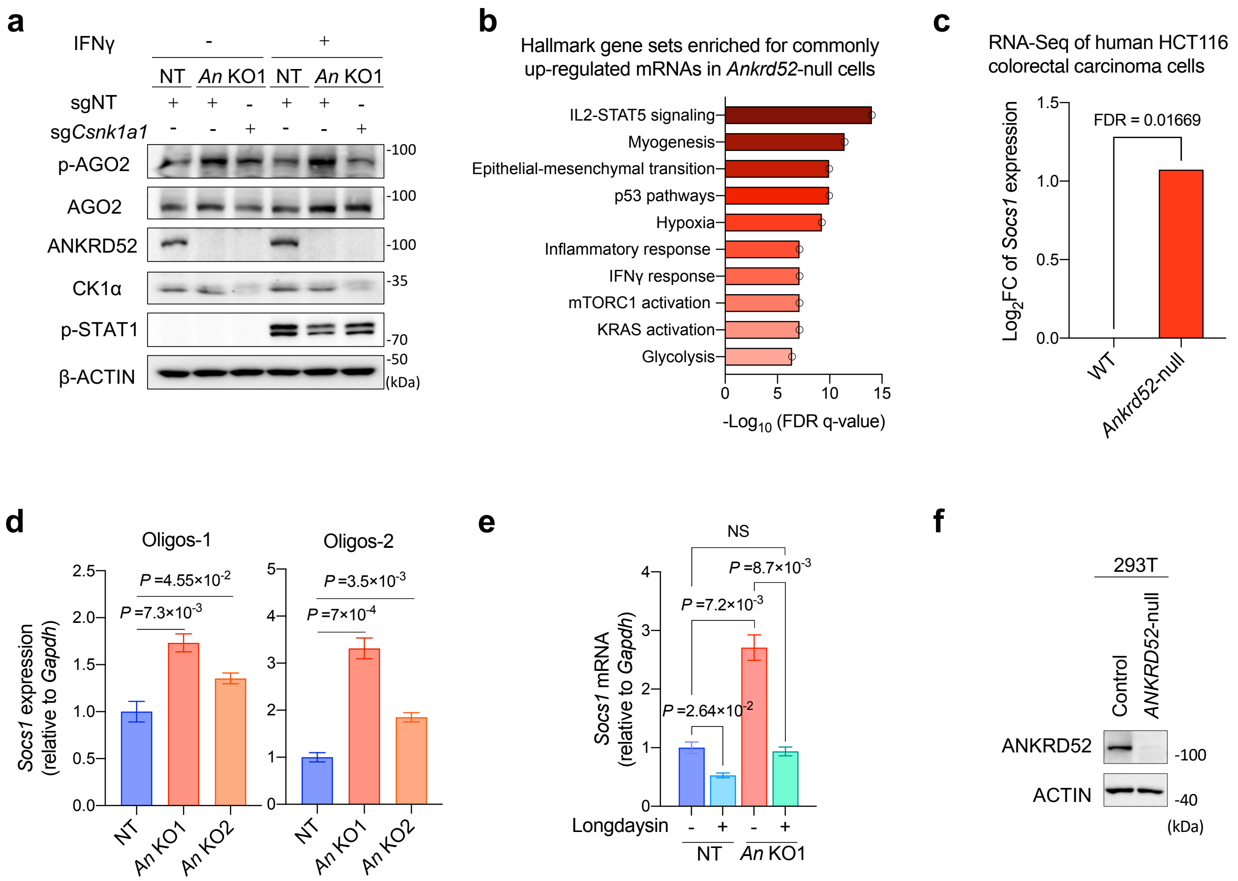

Supplementary Fig. 10 | AGO2 phosphorylation cycle regulates IFNγ response via miRNA-mediated silencing of *Socs1*. Related to Fig. 6. Source data are provided as a Source Data file.

a, p-AGO2 and p-STAT1 abundance in *Ankrd52*-null MC38 cells with activating or inactivating CK1*a* treated with or without IFNγ (10 ng/ml). Data are representative of two independent experiments.

b, Selected hallmark gene sets enriched for commonly up-regulated mRNAs in *Ankrd52*-null (both *Ankrd52* knockout-1 and knockout-2, 1.5-fold-change cut-off, $P < 0.05$, analyzed by edgeR) MC38 cells compared to control cells ($n = 2$ replicates for NT, *Ankrd52* knockout-1 and knockout-2 in RNA-Seq).

c, Log_2FC of *SOCS1* mRNA level in RNA-Seq analysis of *ANKRD52*-null HCT116 cells compared to WT parental cells reported by Golden *et al.*, *Nature*, 2017. HCT116, a human colorectal carcinoma cell line.

d, *Socs1* mRNA level in control and *Ankrd52*-null MC38 cells ($n = 3$ replicates per group). Mean ± s.e.m., two-tailed unpaired Student's *t*-test.

e, *Socs1* mRNA level in control and *Ankrd52*-null MC38 cells treated with Londaysin (200 μM) for 24 hours ($n = 3$ replicates per group per condition). Mean ± s.e.m., two-tailed unpaired Student's *t*-test.

f, Protein abundance of ANKRD52 in control and *ANKRD52*-null 293T cells. Data are representative of two independent experiments.

Supplementary Fig. 11

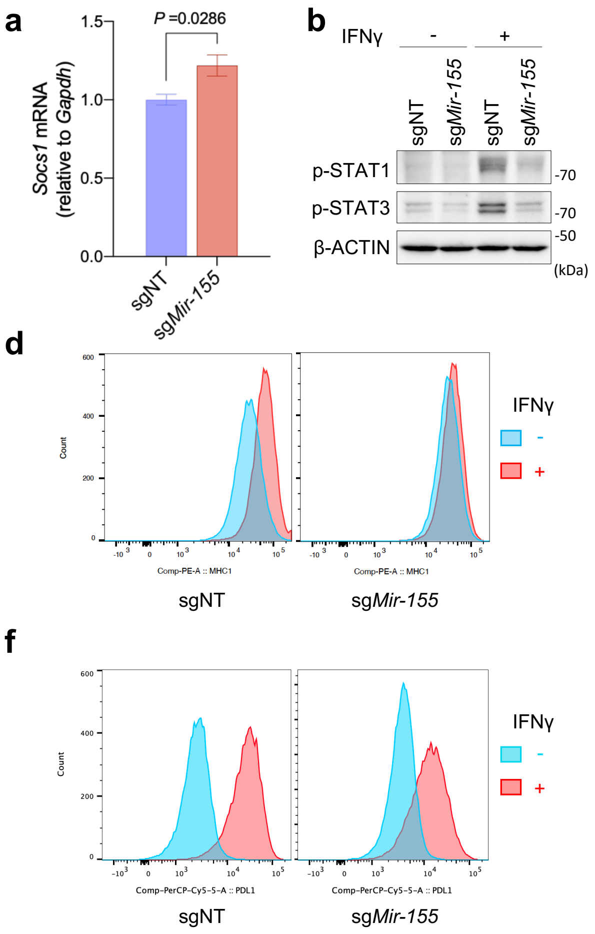

Supplementary Fig. 11 | miR-155 inactivation down-regulates IFNγ response via miRNA-mediated silencing of *Socs1*. Related to Fig. 6. Source data are provided as a Source Data file.

a, *Socs1* mRNA level in MC38 cells with inactivated miR-155 (n = 5 per group). Mean ± s.e.m., two-tailed unpaired Student's t-test.

b, p-STAT1 and p-STAT3 abundance in MC38 cells with inactivated miR-155 treated with IFNγ. Data are representative of two independent experiments.

c, Killing of OVA-treated MC38 cells with inactivated miR-155 by OT-I T cells (n = 3 per group). Mean ± s.e.m., two-way ANOVA and multiple two-tailed unpaired Student's t-test.

d, **e**, Membrane MHC-1 expression in MC38 cells with inactivated miR-155 after IFNγ treatment. MFI of $H2\text{-}K^b$ was normalized by the responding group without IFNγ treatment (n = 3 per group per condition). Mean ± s.e.m., two-tailed unpaired Student's t-test.

f, **g**, Membrane PD-L1 expression in MC38 cells with inactivated miR-155 after IFNγ treatment (n = 3 per group per condition). Mean ± s.d., two-tailed unpaired Student's t-test.

Supplementary Fig. 12

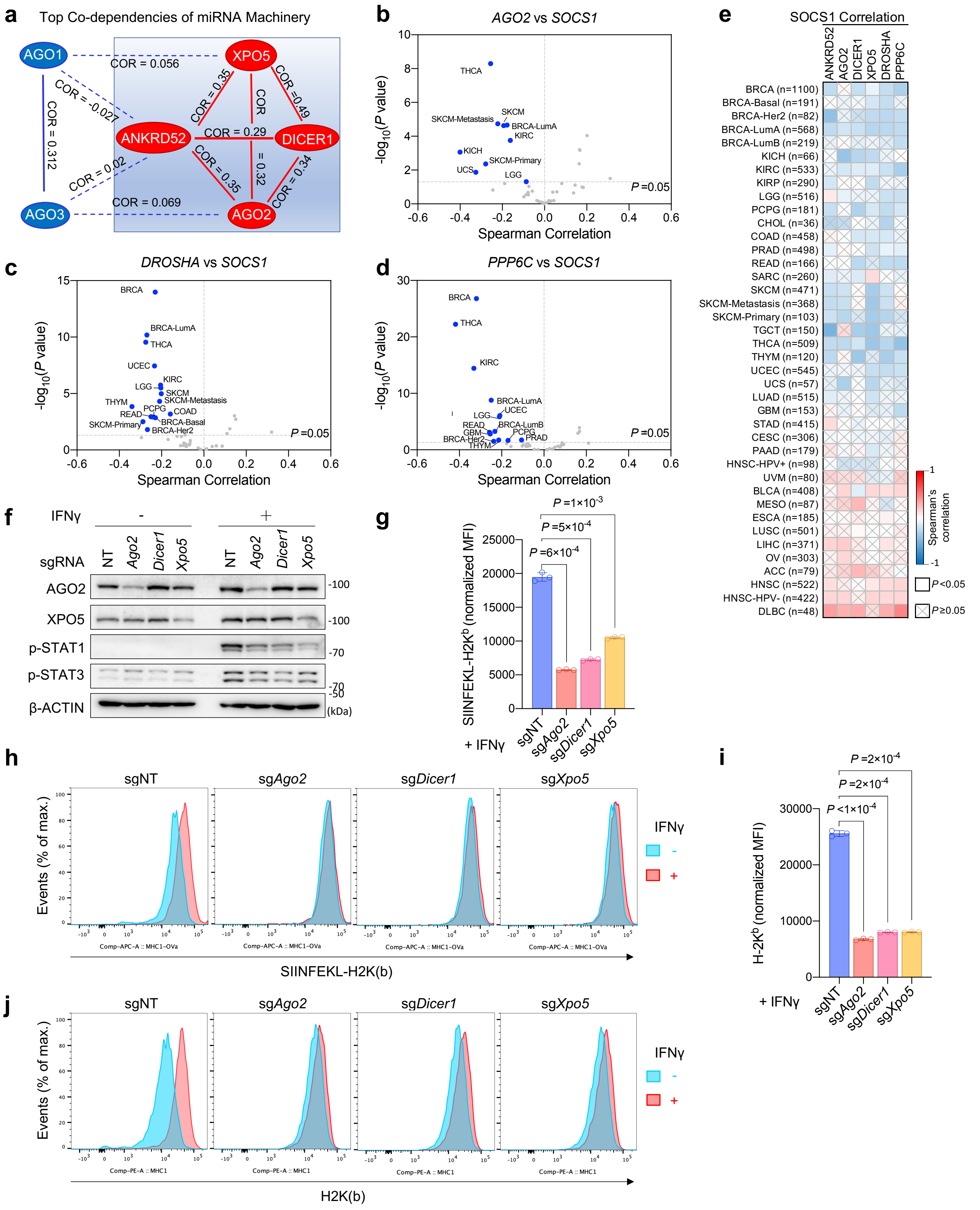

Supplementary Fig. 12 | The core miRNA machinery is required for IFNγ and T cell response. Related to Fig. 7. Source data are provided as a Source Data file.

a, Diagram showing the Spearman's correlation of co-dependency of miRNA machinery components in CRISPR (Avana) Public 20Q3 database. Solid lines depict significant positive correlations ($P < 0.001$) and dashed lines depict weak correlation.

b–**d**, Volcano plot showing the Spearman's correlation and estimated significance of *AGO2* (**b**), *DROSHA* (**c**) or *PPP6C* (**d**) with *SOCS1* mRNA levels from RNA-seq data across all TCGA cancer types. Each dot represents a cancer type in TCGA; blue dots indicate significant negative correlations ($P < 0.05$, analyzed by TIMER).

e, Heatmap showing the Spearman's correlation and estimated significance of *ANKRD52*, *AGO2*, *DICER1*, *XPO5*, *DROSHA* or *PPP6C* with *SOCS1* mRNA levels across all TCGA cancer types.

f, p-STAT1 and p-STAT3 abundance in MC38 cells with targeted sgRNAs for *Ago2*, *Dicer1* or *Xpo5* after IFNγ treatment. Data are representative of two independent experiments.

g, **h**, SIINFEKL-H2K^b presentation in OVA-treated MC38 cells with targeted deletions. MFI of SIINFEKL-H2K^b was normalized by the responding group without IFNγ treatment ($n = 3$ per group per condition). Data are representative of three independent experiments and represented as mean ± s.d., significance was determined using two-tailed unpaired Student's *t*-test.

i, **j**, Membrane MHC-1 expression in MC38 cells with inactivated miRNA machinery component after IFNγ treatment. MFI of H2-K^b was normalized by the responding group without IFNγ treatment ($n = 3$ per group per condition). Mean ± s.d., two-tailed unpaired Student's *t*-test.

Supplementary Fig. 13

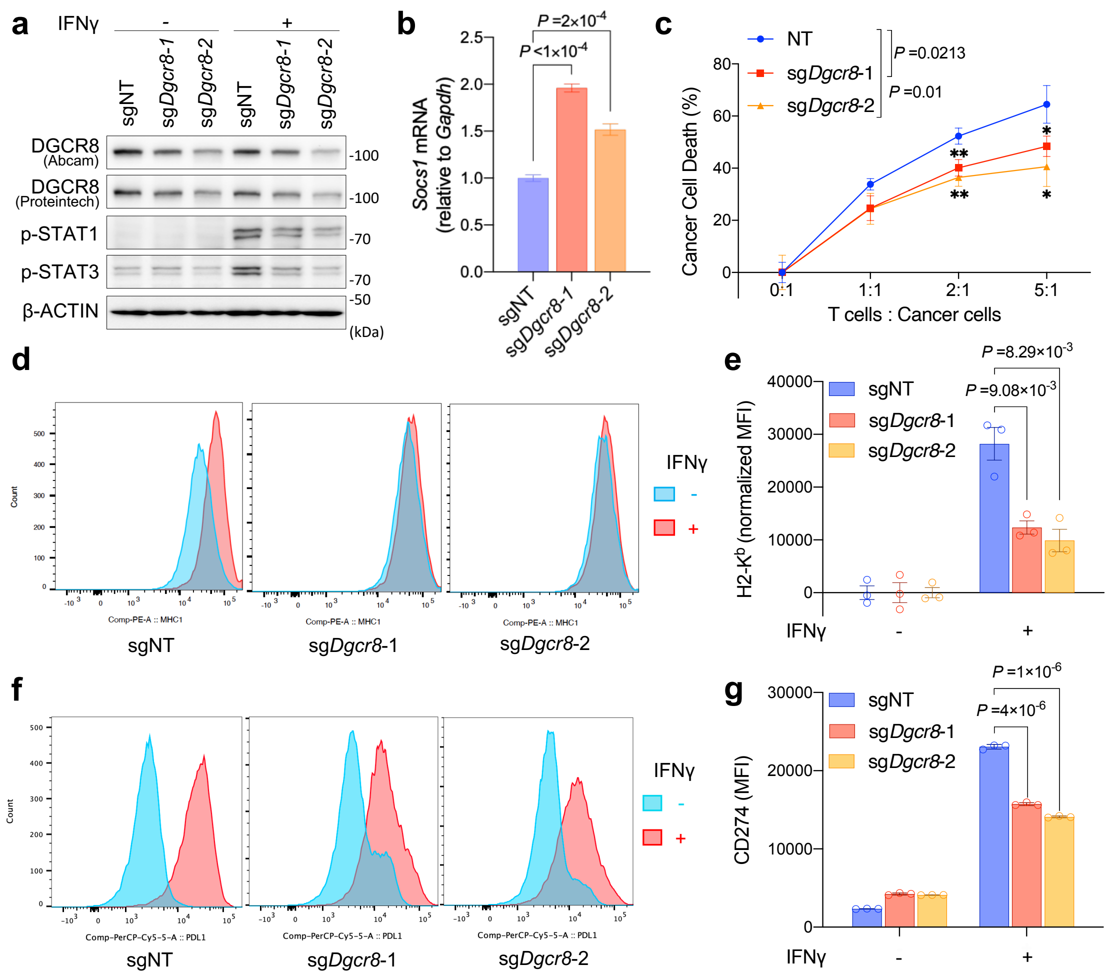

Supplementary Fig. 13 | DGCR8 is required for IFNγ and T cell response. Related to Fig. 7. Source data are provided as a Source Data file.

a, DGCR8, p-STAT1 and p-STAT3 abundance in MC38 cells harboring *Dgcr8* sgRNAs treated with IFNγ. Data are representative of three independent experiments.

b, *Socs1* mRNA level in MC38 cells with inactivated *Dgcr8* ($n = 5$ per group). Data are representative of two independent experiments. Mean ± s.e.m., two-tailed unpaired Student's *t*-test.

c, Killing of OVA-treated MC38 cells with inactivated *Dgcr8* by OT-I T cells ($n = 3$ per group). Mean ± s.e.m., two-way ANOVA and multiple two-tailed unpaired Student's *t*-test.

d, **e**, Membrane MHC-1 expression in MC38 cells with inactivated *Dgcr8* after IFNγ treatment. MFI of H2-K^b was normalized by the responding group without IFNγ treatment ($n = 3$ per group per condition). Mean ± s.e.m., two-tailed unpaired Student's *t*-test.

f, **g**, Membrane PD-L1 expression in MC38 cells with inactivated *Dgcr8* after IFNγ treatment (*n* = 3 per group per condition). Mean ± s.d., two-tailed unpaired Student's *t*-test.

Supplementary Fig. 14

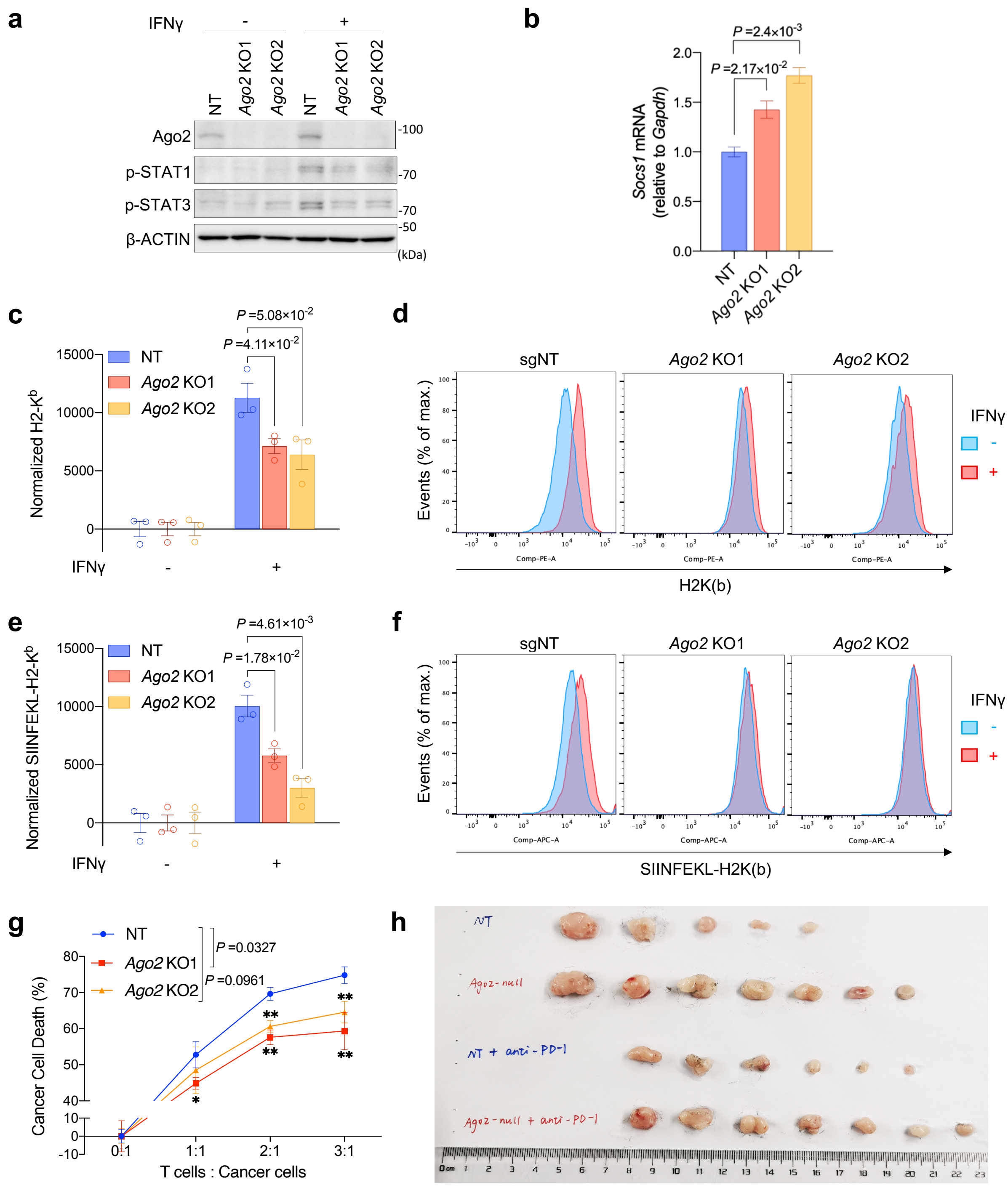

Supplementary Fig. 14 | AGO2 is required for IFNγ and T cell response. Related to Fig. 7. Source data are provided as a Source Data file.

a, p-STAT1 and p-STAT3 abundance in *Ago2*-null MC38 cells treated with IFNγ. Data are representative of three independent experiments.

b, *Socs1* mRNA level in *Ago2*-null MC38 cells (*n* = 3 per group). Mean ± s.e.m., two-tailed unpaired Student's *t*-test.

c, **d**, Membrane MHC-1 expression in in *Ago2*-null MC38 cells after IFNγ treatment. MFI of H2-K^b was normalized by the responding group without IFNγ treatment (*n* = 3 per group per condition). Mean ± s.e.m., two-tailed unpaired Student's *t*-test.

e, **f**, Presentation of OVA-derived peptide (SIINFEKL) in OVA-treated control and *Ago2*-null MC38 cells. MFI of SIINFEKL-H2K^b was normalized by the responding group without IFNγ treatment (*n* = 3 per group per condition). Mean ± s.e.m., two-tailed unpaired Student's *t*-test.

g, Killing of OVA-treated *Ago2*-null MC38 cells by OT-I T cells (*n* = 3 per group). Mean ± s.e.m., two-way ANOVA and two-tailed unpaired Student's *t*-test.

h, *Ago2*-null (*n* = 7 per group) or control (*n* = 5 for vehicle treatment and *n* = 6 for PD-1 antibody treatment) MC38 tumors grown in WT mice treated with PD-1 antibody or not.

Supplementary Fig. 15

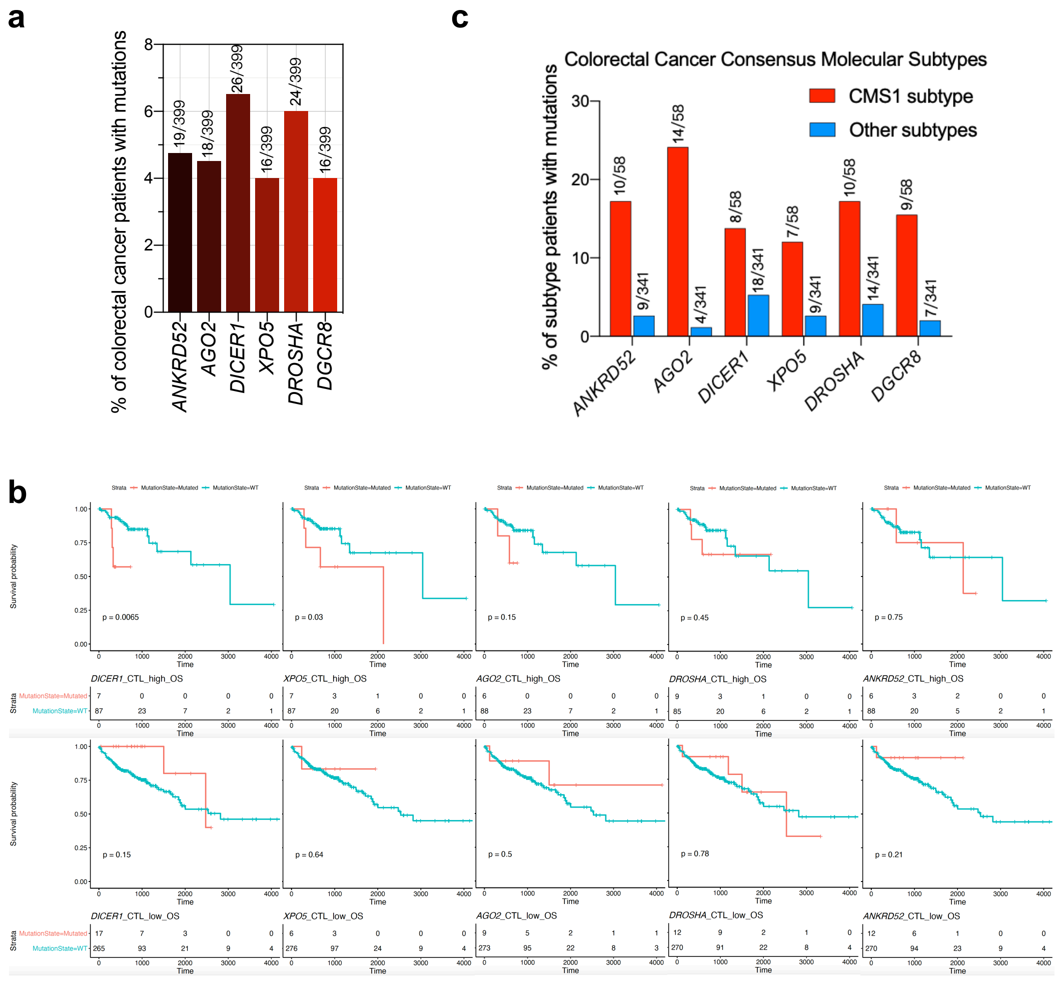

Supplementary Fig. 15 | Mutations in miRNA machinery are associated with T cell mediated anti-tumor immunity in colorectal cancer patients. Related to Fig. 7. Source data are provided as a Source Data file.

a, Bar plot showing the percentage of patients with mutations in miRNA machinery across colon adenocarcinoma (COAD) in TCGA datasets.

b, Correlation of miRNA machinery mutations with survival of COAD patients depending on calculated level of cytotoxic T cell infiltration (Log-rank test). All patients in the TCGA COAD study were divided according to mutations of each component of miRNA machinery.

c, Bar plot showing the percentage of patients with mutations in miRNA machinery across CMS subtypes of COAD.
