# Supplementary material for: Tumor evolution selectively inactivates the core microRNA machinery for immune evasion
Source: Nat Commun. 2021 Dec 1;12:7003. doi: 10.1038/s41467-021-27331-3 (PMC8636623; doi:10.1038/s41467-021-27331-3)
Supplement: Supplementary file 7 — Description of Additional Supplementary Files [file 41467_2021_27331_MOESM7_ESM.pdf]

Title: Supplementary Data 1:
Description: sgRNA information and performance in the *in vivo* CRISPR screen using analysis of edgeR.

Title: Supplementary Data 2:
Description: sgRNA information and performance in the *in vitro* CRISPR screen using analysis of edgeR.

Title: Supplementary Data 3:
Description: Significant differentially expressed genes in *Ankrd52*-null MC38 cells after treatment with IFNγ or not using analysis of edgeR.

Title: Supplementary Data 4:
Description: sgRNA sequences used in the research.

Title: Supplementary Data 5:
Description: qPCR primers used in the research.
