# Supplementary material for: Tumor evolution selectively inactivates the core microRNA machinery for immune evasion
Source: Nat Commun. 2021 Dec 1;12:7003. doi: 10.1038/s41467-021-27331-3 (PMC8636623; doi:10.1038/s41467-021-27331-3)
Supplement: Supplementary file 8 — Reporting Summary [file 41467_2021_27331_MOESM8_ESM.pdf]

nature research

Corresponding author(s): Yong Cang

Last updated by author(s): Oct 28, 2021

Reporting Summary

Nature Research wishes to improve the reproducibility of the work that we publish. This form provides structure for consistency and transparency in reporting. For further information on Nature Research policies, see our Editorial Policies and the Editorial Policy Checklist.

Statistics

For all statistical analyses, confirm that the following items are present in the figure legend, table legend, main text, or Methods section.

n/a	Confirmed	
☐	☒	The exact sample size (*n*) for each experimental group/condition, given as a discrete number and unit of measurement
☐	☒	A statement on whether measurements were taken from distinct samples or whether the same sample was measured repeatedly
☐	☒	The statistical test(s) used AND whether they are one- or two-sided<br>*Only common tests should be described solely by name; describe more complex techniques in the Methods section.*
☒	☐	A description of all covariates tested
☐	☒	A description of any assumptions or corrections, such as tests of normality and adjustment for multiple comparisons
☐	☒	A full description of the statistical parameters including central tendency (e.g. means) or other basic estimates (e.g. regression coefficient) AND variation (e.g. standard deviation) or associated estimates of uncertainty (e.g. confidence intervals)
☐	☒	For null hypothesis testing, the test statistic (e.g. *F*, *t*, *r*) with confidence intervals, effect sizes, degrees of freedom and *P* value noted<br>*Give P values as exact values whenever suitable.*
☒	☐	For Bayesian analysis, information on the choice of priors and Markov chain Monte Carlo settings
☐	☒	For hierarchical and complex designs, identification of the appropriate level for tests and full reporting of outcomes
☐	☒	Estimates of effect sizes (e.g. Cohen's *d*, Pearson's *r*), indicating how they were calculated

Our web collection on statistics for biologists contains articles on many of the points above.

Software and code

Policy information about availability of computer code

Data collection	The somatic mutation data inferred by MuTect2 for 33 TCGA cohorts were downloaded from UCSC Xena (https://gdc.xenahubs.net) with R package UCSCXenaTools. All of TCGA analysis data (containing the results of EPIC and TIMER analysis) were obtained from TIMER2.0 database (http://timer.cistrome.org).
Data analysis	For RNASeq, FastQC (0.11.2) was used to generate QC report and the adapters of each sample were trimmed, respectively.<br>To measure gene expression level, fastq file of each sample was mapped to reference genome to get reads count table as input for differential expression analysis. The clean reads were mapped to the mouse (Mus musculus) genome (mm10) using STAR (2.4.2a) and annotated with transcriptome database (genecode vM13). Gene abundance estimation by read counts was conducted with the software RSEM (1.2.29). Normalized tags per million (TPM) were calculated on the number of clean reads mapped to specific region of genome using the relative log expression (RLE) method in edgeR (3.16.5). Differentially expressed genes (DEG) refer to compare gene expression level between two samples or two groups. DEG were defined by using the criterion that fold change > 1.5 and adjust P < 0.05 (adjust P-value). Pathway enrichment analysis (GO and KEGG) on DEGs was performed on differentially expressed genes using GOstats (2.40.0) in R, with threshold that P < 0.01. Gene set enrichment analysis for molecular signature was carried out using GSEA software with threshold that P < 0.01. Tumor infiltrating lymphocytes were analyzed by mMCP counter.<br>For mutation calling, To detect mutations (SNP and InDel) in RNA-seq datasets from tumors under different immune selection, we used the aligner STAR (2.5.2b) to map the reads to the genome (GRCm38 - mm10) and used MarkDuplicates in picard-tools-1.94 (http://broadinstitute.github.io/picard) to dedup the bam file. And then we applied joint call to detect SNP and Indels across all RNA-seq datasets by following the GATK (3.7.0) best-practice guidelines (https://gatk.broadinstitute.org/hc/en-us/sections/360007226651-Best-Practices-Workflows). In the variant filtering step, we used specific hard filter to get the filtered VCF file. Then we used ANNOVAR to annotate the mutation sites.<br>For CRISPR screen, MAGeCKFlute was used to generate read counts for each sgRNA based on fastq files from paired-end NGS of PCR amplicons. Tumor-intrinsic immune evasion gene hits were determined based on both integrated beta score ranking (generated by MAGeCK-

RRA) and log2 fold-change (generated by edgeR) with relevant P value (< 0.05).
BD LSRFortessa X-20 flow cytometry system was used for FACs data acquisition and FlowJo (10.4) was used for data analysis.
Microsoft Excel (16.27) was used to organize data into tables. In all cases, ****P < 0.0001, ***P < 0.001, **P < 0.01, *P < 0.05. Statistical analyses were performed using Graph-Pad Prism (8.2.1) or the R language (3.6.0) programming environment using RStudio (1.2.1335).

For manuscripts utilizing custom algorithms or software that are central to the research but not yet described in published literature, software must be made available to editors and reviewers. We strongly encourage code deposition in a community repository (e.g. GitHub). See the Nature Research guidelines for submitting code & software for further information.

Data

Policy information about availability of data

All manuscripts must include a data availability statement. This statement should provide the following information, where applicable:

- Accession codes, unique identifiers, or web links for publicly available datasets
- A list of figures that have associated raw data
- A description of any restrictions on data availability

The raw FASTQ files and the source datasets generated and analyzed in this study for the sequencing data are available in the Genome Sequence Archive (GSA) database upon accession number (CRA004140, CRA004141, CRA004145, CRA004146). Descriptions of the analyses, tools and algorithms are provided in the Methods or GSA. Source data are provided with this paper.

Field-specific reporting

Please select the one below that is the best fit for your research. If you are not sure, read the appropriate sections before making your selection.

[x] Life sciences [ ] Behavioural & social sciences [ ] Ecological, evolutionary & environmental sciences

For a reference copy of the document with all sections, see nature.com/documents/nr-reporting-summary-flat.pdf

Life sciences study design

All studies must disclose on these points even when the disclosure is negative.

Sample size	For CRISPR screens, the mutagenized cell pool was guaranteed with at least 1000x coverage of the sgRNA library volume in every step of. Using these sample sizes allowed for the application of robust statistical assessments for each experiment or analysis.<br>The sample sizes were chosen according to the convention in the field. Group sizes for in vivo validation experiments were selected empirically based upon prior knowledge of the intragroup variation of tumor challenges and immunotherapy treatment.<br>For small-scale experiments, the number of replicates exceeds at least 3 technical replicates repeated measurements of the same original sample).
Data exclusions	No data were excluded from any experiments and figures shown.
Replication	Replicates were used in all experiments as noted in text, figure legends and methods. For CRISPR screens, 10 sgRNAs per gene, timepoints, at least 5 tumors, high library coverage and sample correlations for each group were used for reproducibility improvement. All in vivo experiments were repeated at least twice with consonant results, with the exception of those that were supporting/confirmatory in nature and appear ONLY in Extended Data (e.g. SizeMatch experiment). All experiments presented for which replication was attempted were successfully replicated.
Randomization	Mice were age-matched and randomized where appropriate (e.g. prior to initiating treatment for matched conditions).<br>For in vitro experiments, cells are randomized planted for treatment and randomized collected.
Blinding	No blinding was performed in the in vivo experiments due to requirements for cage labeling and staffing needs.<br>No blinding was performed in the in vitro experiments due to requirements for distinguishing gene-edited cells from control cells.

Reporting for specific materials, systems and methods

We require information from authors about some types of materials, experimental systems and methods used in many studies. Here, indicate whether each material, system or method listed is relevant to your study. If you are not sure if a list item applies to your research, read the appropriate section before selecting a response.

Materials & experimental systems

n/a	Involved in the study
	☒ Antibodies
	☒ Eukaryotic cell lines
☒	☐ Palaeontology and archaeology
	☒ Animals and other organisms
☒	☐ Human research participants
☒	☐ Clinical data
☒	☐ Dual use research of concern

Methods

n/a	Involved in the study
☒	☐ ChIP-seq
	☒ Flow cytometry
☒	☐ MRI-based neuroimaging

Antibodies

Antibodies used

For western blot, primary antibodies against mouse ANKRD52 (Santa Cruz, sc-398544), human ANKRD52 (Bethyl, A302-372A), OVA (Sigma, SAB5300165), β-Actin (CST, 4967), JAK1 (CST, 3332), p-STAT1 (Tyr701) (CST, 9167), p-STAT3 (Tyr705) (CST, 9145), SOCS3 (CST, 52113), TAP1 (CST, 12341), FLAG (Sigma, F3165), Vinculin (Sigma, v9131), CK1a (Santa Cruz, sc-6477), PPP6C (Abcam, EPR8764), AGO2 (CST, 2897S), XPO5 (CST, 12565S) and DGCR8 (Abcam, ab191875; Proteintech, 10996-1-AP) were used.
For flow cytometry, the following anti-mouse fluorochrome-conjugated antibodies were used: AF700 anti-CD45 (560510), PerCP-Cy5.5 anti-CD4 (550954), BV510 anti-CD8 (563068) and BV605 anti-CD44 (563058), BV605 anti-CD25 (563061) purchased from BD Biosciences; PE anti-H2Kb/H2Db (114607), APC anti-H2Kb bound to SIINFEKL (141605), FITC anti-Lag-3 (369308), FITC anti-CD69 (104506) and AF594 anti-GZMB (372216) purchased from BioLegend; PerCP-Cy5.5 anti-CD274 (46-5982-82), PE-Cy7 anti-FoxP3 (25-5773-82) and PE anti-Tim-3 (12-5870-82) purchased from ebioscience.

Validation

For western blot, primary antibodies against ANKRD52 (Santa Cruz, sc-398544), OVA (Sigma, SAB5300165), β-Actin (CST, 4967), JAK1 (CST, 3332), p-STAT1 (Tyr701) (CST, 9167), p-STAT3 (Tyr705) (CST, 9145), SOCS3 (CST, 52113), TAP1 (CST, 12341), FLAG (Sigma, F3165), Vinculin (Sigma, v9131), PPP6C (Abcam, EPR8764), AGO2 (CST, 2897S) and XPO5 (CST, 12565S) were validated by mouse cell samples. Human ANKRD52 (Bethyl, A302-372A) was validated by human cell samples. These antibodies were diluted as 1:500 for WB.
For flow cytometry, the following anti-mouse fluorochrome-conjugated antibodies were validated using mouse tumor or cell line samples: AF700 anti-CD45 (560510), PerCP-Cy5.5 anti-CD4 (550954), BV510 anti-CD8 (563068) and BV605 anti-CD44 (563058), BV605 anti-CD25 (563061) purchased from BD Biosciences; PE anti-H2Kb/H2Db (114607), APC anti-H2Kb bound to SIINFEKL (141605), FITC anti-Lag-3 (369308), FITC anti-CD69 (104506) and AF594 anti-GZMB (372216) purchased from BioLegend; PerCP-Cy5.5 anti-CD274 (46-5982-82), PE-Cy7 anti-FoxP3 (25-5773-82) and PE anti-Tim-3 (12-5870-82) purchased from ebioscience. These antibodies were diluted as 1:100 for FACs.

Eukaryotic cell lines

Policy information about cell lines

Cell line source(s)

293FT (R70007) and 293T (/17) (CRL-11268) cell lines were originally purchased from Thermo Fisher and ATCC respectively and cultured according to the manufacturers' manual specifically. MC38 cell line was provided by WuXi AppTec and cultured using DMEM (Gibco) with 10% fetal bovine serum (FBS) respectively.
MC38-OVA cell lines were constructed by introducing cDNA of OVA peptide into plenti6.3 vector (Thermo Fisher, K533000). The cDNA sequences for OVA peptide (p.257-264, SIINFEKL) were synthesized at GENEWIZ. Blasticidin (Gibco, A1113903) selection was used for lentivirus transduced cells (MOI ≈ 0.3) to generate OVA stably expressing cells.
OT-I T cells were isolated from spleen and lymph nodes of OT-I transgenic mouse (a gift from Bing Sun's Lab at SIBCB) by EasySep™ Mouse CD8+ T Cell Isolation Kit (STEMCELL, 19753). Fresh isolated CD8+ T cells were cultured in complete T cell medium (RPMI-1640 with 10%FBS, 20 mM HEPES, 1 mM sodium pyruvate, 0.05 mM 2-mercaptoethanol, 2 mM L-glutamine, 50 U/ml streptomycin and penicillin) and treated with 2 ng/mL recombinant mouse IL-2 (Novoprotein, CK24).

Authentication

None of the cell lines were independently authenticated.

Mycoplasma contamination

All cell lines used in this study were tested as mycoplasma-negative using the Universal Mycoplasma Detection Kit (ATCC, 30-1012K).

Commonly misidentified lines
(See ICLAC register)

None.

Animals and other organisms

Policy information about studies involving animals; ARRIVE guidelines recommended for reporting animal research

Laboratory animals

C57BL/6J and Foxn1nu (nude) mice (female, aged at 6-8 weeks and weighing approximately 18-22g) were used for tumor implantation experiments and purchased from Shanghai SLAC Laboratory Animal. OT-I transgenic mouse was a gift from Bing Sun's Lab at SIBCB. OT-I transgenic mouse (male or female, aged at 6-10 weeks) were used to provide fresh CD8+ T cells.

Wild animals

Wild animals were not used in this study.

Field-collected samples	Field-collected samples were not used in this study.
Ethics oversight	The protocol and any amendment(s) or procedures involving the care and use of animals in this study were in accordance with Shanghai Institutional Animal Care and Use Committee (IACUC) guidelines and approved by IACUC of ShanghaiTech University or WuXi AppTec. Animal experiments were conducted at the National Facility for Protein Science or OIU of WuXi AppTec.

Note that full information on the approval of the study protocol must also be provided in the manuscript.

Flow Cytometry

Plots

Confirm that:

- [x] The axis labels state the marker and fluorochrome used (e.g. CD4-FITC).
- [x] The axis scales are clearly visible. Include numbers along axes only for bottom left plot of group (a 'group' is an analysis of identical markers).
- [x] All plots are contour plots with outliers or pseudocolor plots.
- [x] A numerical value for number of cells or percentage (with statistics) is provided.

Methodology

Sample preparation	For antigen presentation test, OVA treated (1 µM for 2hr) MC38 cells were simulated with 10 ng/ml IFNγ (CST, 39127) for 24 hr. Then tumor cells were trypsinized and washed in PBS + 2% FBS, stained with antibodies against cell surface H2Kb and H2Kb bound to SIINFEKL as the manufacturer's instructions.<br>For TiLs analysis, tumors were harvested at ~1000 mm3 and cut up on ice for following incubation in collagenase D (1 mg/ml, Roche), DNase I (50 µg /ml, Sigma-Aldrich) and Hyaluronidase (100 µg/mL, Sigma-Aldrich) supplemented RPMI-1640 (Gibco) for 30 min at 37 °C. After incubation, tumor cells were passed through 70-µm filters to remove undigested tumor. Then these single tumor cell suspensions were washed with ice-cold PBS with 2% FBS and stained with Live/Dead (1:1000, Invitrogen) combined with antibodies for 30 min at 4 °C.
Instrument	BD LSRFortessa X-20 flow cytometry system
Software	BD DIVA software used for collection, FlowJo software used for analysis
Cell population abundance	No cell sorting was performed in this manuscript
Gating strategy	All gates were set based on FMO (full-minus one) stains and isotype control antibodies after appropriate compensation using single-stained compensation controls.

- [x] Tick this box to confirm that a figure exemplifying the gating strategy is provided in the Supplementary Information.
